# Reactive Polymeric Membranes for Advanced Water Treatment: Bridging Mechanisms, Kinetics and Scalable Deployment

**DOI:** 10.3390/polym18111387

**Published:** 2026-06-03

**Authors:** Alireza Ranjbari, Soumya Ranjan Mishra, Alireza Pourvahabi Anbari, Philippe M. Heynderickx

**Affiliations:** 1 Center for Green Chemistry and Environmental Biotechnology, Ghent University Global Campus, 119-5 Songdomunhwa-Ro, Yeonsu-Gu, Incheon 21985, Republic of Korea; soumyaranjan.mishra@ghent.ac.kr (S.R.M.); alireza.pourvahabi@ghent.ac.kr (A.P.A.); 2 Department of Green Chemistry and Technology, Faculty of Bioscience Engineering, Ghent University, 653 Coupure Links, B-9000 Ghent, Belgium; 3 Department of Chemistry, Faculty of Science, Ghent University, B-9000 Ghent, Belgium

**Keywords:** reactive polymeric membranes, oxidative filtration, wastewater treatment, redox-active polymers, conductive polymer membranes, membrane fouling, reactive transport kinetics, digital twins, life cycle assessment, upscaling

## Abstract

Reactive polymeric membranes are emerging as promising platforms for advanced water and wastewater treatment because they combine separation with in situ contaminant transformation. Unlike conventional membranes, which mainly retain pollutants, reactive polymeric membranes can enrich, activate, and degrade micropollutants during permeation through built-in radical, redox-active, conductive, or porous catalytic domains. This review discusses the development of intrinsic reactive polymer membranes for oxidative filtration, with emphasis on the links between polymer structure, transport behavior, reactive oxygen species generation, and degradation pathways. Key membrane classes are discussed, including stable-radical polymers, redox-active polymer networks, conductive polymer membranes, and porous conjugated polymer catalytic layers. The review also highlights the importance of reactive transport kinetics, including convection–diffusion–reaction coupling, residence time, Damköhler and Péclet numbers, and adsorption-enhanced degradation. Challenges such as fouling, polymer aging, leaching, byproduct formation, and toxicity-aware benchmarking are discussed within a broader roadmap for technology translation. The review identifies the grand challenges and milestone-based priorities for developing and deploying reactive polymer membranes, including performance targets, standardized reporting, realistic water matrices, scale-up, technology readiness levels, techno-economic analysis, life cycle assessment, artificial intelligence, and digital twins. Together, these elements guide the translation of reactive polymer membrane systems from laboratory research toward full-scale water treatment applications.

## 1. Introduction

Water and wastewater streams are becoming increasingly complex. In addition to conventional pollutants such as suspended solids, organic matter, nitrogen, and phosphorus, modern waters often contain a wide range of low-concentration but high-impact contaminants, including pharmaceuticals, personal care products, pesticides, dyes, and per- and polyfluoroalkyl substances (PFAS) [1,2,3]. Many of these compounds are not effectively removed by biological treatment or conventional low-pressure membrane processes, which are mainly designed for particulate and macromolecular separation [4,5].

At the same time, regulatory frameworks are evolving to address these emerging contaminants. In Europe and the United States, new requirements for micropollutant and PFAS monitoring and removal are increasing the need for advanced treatment technologies that can operate reliably under real water conditions [6]. These developments highlight a key challenge: treatment systems must not only achieve high removal efficiency, but also maintain stability, scalability, and performance in complex and variable water matrices [4,7].

Polymeric membranes are already widely used in water treatment because of their compact design and strong separation performance [8]. Processes such as nanofiltration and reverse osmosis can achieve high rejection of many contaminants [4]. However, separation alone has important limitations when targeting trace organic compounds. First, membranes often transfer contaminants from the treated water to a concentrated reject stream, creating additional challenges for disposal or further treatment [9]. Second, membrane systems are prone to fouling and performance decline, especially in wastewater applications, which increases operational costs and reduces long-term efficiency [10]. Third, rejection-based approaches do not address transformation products or toxicity, and their performance can vary depending on water composition [11].

The aforementioned limitations have motivated the development of systems that combine separation with chemical transformation. One promising approach is oxidative filtration, where contaminants are degraded during membrane passage rather than only being retained. In this concept, the membrane acts not only as a separation barrier but also as a reactive medium [12,13]. Pollutants can be enriched near reactive domains, transported through the membrane structure, and degraded by catalytic or redox-active sites. This integration allows treatment and transformation to occur in a single step, which can reduce system complexity and improve overall efficiency [14].

However, oxidative filtration introduces new challenges. The residence time of water inside a membrane is typically very short, often on the order of milliseconds to seconds. As a result, reaction rates must be sufficiently fast, and mass transfer must be carefully managed [1]. In addition, real water matrices contain natural organic matter and inorganic ions that can compete for reactive species, affect transport, and influence reaction pathways [15]. These factors make it necessary to consider not only material properties but also transport phenomena and reaction kinetics when designing reactive membranes [1].

Recent research has increasingly focused on intrinsic reactive polymer membranes, where the polymer itself plays an active role in the reaction process [16]. In these systems, reactivity is not only provided by embedded catalysts but is also determined by polymer chemistry and the local microenvironment within the membrane. Functional groups, pore structure, hydrophilicity, and charge can all influence pollutant partitioning, reactive species generation, and degradation pathways [17]. This approach opens new opportunities for designing membranes that combine separation, transport control, and chemical reactivity in a single material platform [18].

The growing interest in these systems is driven not only by scientific advances but also by practical needs. Regulatory pressure for micropollutant removal, concerns about concentrate management, and the demand for energy-efficient treatment technologies are all pushing the field toward integrated solutions. To move from laboratory studies to real applications, it is essential to understand how material properties, transport processes, and reaction mechanisms interact under realistic conditions.

The scope of this review targets polymer-based reactive membrane systems in which the polymer structure, functional groups, redox activity, conductivity, or polymer-defined pore environment contributes directly to contaminant transformation during filtration. The review includes stable-radical polymers, redox-active polymers, conductive polymer membranes, and porous polymer catalytic layers used for oxidative filtration or closely related flow-through treatment. Conventional membranes used only for passive separation, purely inorganic catalytic membranes, and bulk AOP reactors without a membrane-integrated polymer function are not the main focus. This scope allows the discussion to center on how polymer chemistry, membrane transport, ROS pathways, and practical deployment challenges are connected.

While several reviews have discussed catalytic membranes, photocatalytic membrane reactors, oxidative filtration, and advanced oxidation processes, this review focuses on reactive polymeric membranes for oxidative filtration, with emphasis on the links between polymer design, transport phenomena, and reaction kinetics. It covers different classes of reactive polymers, including radical-based systems, redox-active polymers, conductive membranes, and porous catalytic layers. The review also discusses modeling approaches for coupled transport and reaction, standardized reporting methods, scale-up considerations, and emerging tools such as machine learning and digital twins for process optimization. Finally, this review outlines key challenges and future directions for translating reactive membrane concepts into practical water treatment technologies.

## 2. Classes of Intrinsic Reactive Polymer Membranes

### 2.1. Radical Polymer Membranes

Persistent free radicals incorporated into polymer networks represent a unique pathway for achieving in situ oxidation during filtration. In these systems, the polymer itself carries stable unpaired electrons (for example, through nitroxide radicals, conjugated carbon-backbone radicals, or other radical centers) that can directly abstract hydrogen from organic pollutants. This hydrogen abstraction step converts the polymer radical (R^•^) into a polymer-bound hydrogen (RH), while generating a carbon-centered or peroxyl radical on the pollutant. Crucially, molecular oxygen in the water can react with the polymer-bound hydrogen to regenerate the radical state, effectively closing a self-sustaining cycle [19]. This mechanism was demonstrated in a recent study: a radical-containing polymer network filtered water and degraded sulfamethoxazole (SMX) by first forming peroxyl radicals, which then gave rise to hydroxyl radicals in situ. Importantly, the carbon-centered radicals on the polymer were replenished by O_2_, allowing the polymer to remain active [20].

In practice, stable-radical polymer membranes are designed so that (a) the radical centers are long-lived under aqueous conditions, and (b) the polymer framework is robust to maintain permeability and surface area. Typical examples include polymers functionalized with nitroxide groups or aromatic radicals, which are known from organic battery research and catalysis to persist for hours or days. Characterization of such membranes often involves electron spin resonance (ESR/EPR) spectroscopy to verify the presence and longevity of the unpaired electrons, as well as standard chemical analyses (FTIR, XPS) to detect any gradual oxidation of the polymer backbone during operation [21]. Over multiple filtration cycles, one expects to see depletion of radical signature if regeneration is incomplete, and accumulation of oxygenated functional groups (such as ketones or alcohols) on the polymer as aging signatures. Understanding these aging markers is essential to differentiate simple fouling from actual loss of chemical activity. Preliminary data in model systems show that radical polymers can retain activity over tens of hours of continuous operation, but eventual decay of radical density does occur and correlates with slight drops in flux or conversion efficiency. In summary, radical polymer membranes operate by polymer-mediated autoxidation: pollutants are oxidized via H-atom abstraction, and the polymer’s radical state is continuously cycled by O_2_, permitting sustained degradation without adding external catalysts or oxidants [22].

### 2.2. Redox-Active Polymer Mediators

**Figure 1:**
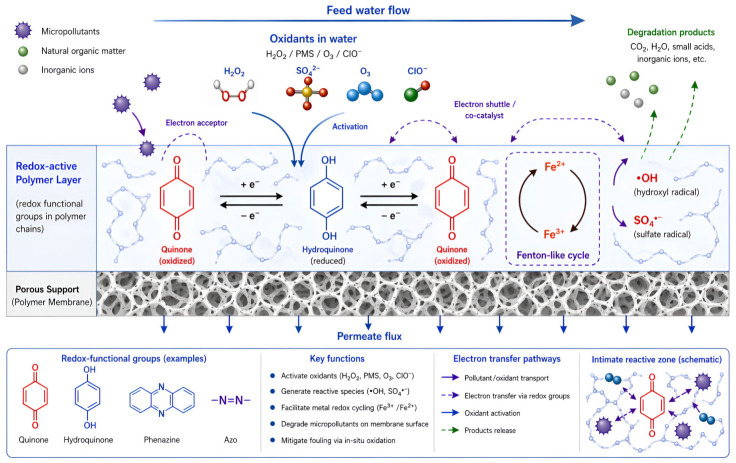
Schematic illustration of a redox-active polymer membrane for oxidative filtration, showing how embedded redox functional groups (e.g., quinone/hydroquinone) mediate electron transfer, activate oxidants (e.g., H_2_O_2_, PMS), and facilitate radical generation (HO^•^, SO_4_^•−^) for in situ degradation of micropollutants during permeation.

A second class of reactive polymers relies on built-in redox functionality rather than free radicals. These redox-active polymer membranes contain functional groups such as quinone/hydroquinone, phenazine, azo, or heteroatom-bearing rings (azines, quinolines) that can reversibly exchange electrons with dissolved species [23]. In an oxidative-filtration context, the polymer serves as an electron shuttle or co-catalyst; it can accept electrons from an oxidant activation process or donate electrons to reduce a metal co-catalyst, thus accelerating the overall redox cycle [24]. For example, a quinone-containing polymer can facilitate Fenton-like chemistry by cycling Fe(III) ⇌ Fe(II) at its surface; the polymer’s redox group temporarily holds an electron, so that Fe^3+^ is reduced back to Fe^2+^ without adding chemical reductants. Alternatively, some redox polymers can directly interact with peroxymonosulfate or H_2_O_2_, abstracting an electron to generate sulfate or hydroxyl radicals while the polymer becomes oxidized. The polymer is then chemically regenerated by ambient reductants in the water (organic matter) or by spontaneous back-reaction. In essence, the polymeric film creates an intimate zone where contaminants and oxidants can undergo fast electron transfer on the polymer surface (Figure 1).

Translating these ideas into membrane form involves embedding redox moieties either along the polymer backbone or as pendants, and ensuring electrical connectivity if needed. For instance, a polyoxometalate-linked polymer membrane was shown to continuously oxidize organics by cycling the polyoxometalate between different oxidation states [25]. Similarly, nitroxide or trityl radicals in polymers can cycle with H_2_O_2_ to produce hydroxyl radicals. In practice, one observes that redox-polymer membranes often have higher pollutant conversion than purely inert membranes under the same conditions, reflecting this catalytic cycle [26]. Characterization of such membranes includes cyclic voltammetry of the dry film (to identify redox peaks of the functional groups) and tracer tests with known oxidants (adding low-dose H_2_O_2_ to see if reaction rate increases). Aging in redox polymers typically manifests itself as a gradual loss of redox capacity, for example, irreversible structural changes in quinone to non-redox-active products, which would show up as decreasing peak currents in voltammograms over cycles. Nonetheless, by selecting robust redox groups (conjugated aromatics rather than unstable sulfur compounds) and minimizing leaching, polymer-assisted electron shuttling can provide extended pollutant-removal performance without the constant addition of sacrificial chemicals [27].

### 2.3. Conductive Polymer Membranes

**Figure 2:**
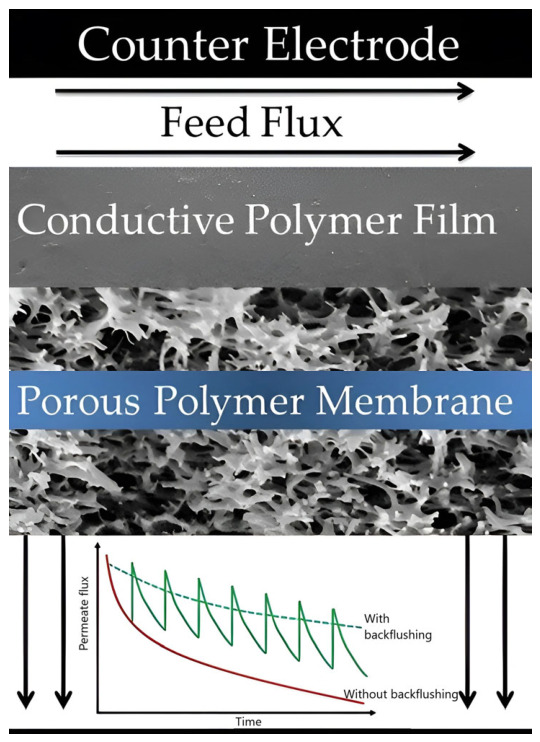
Schematic representation of a conductive polymer membrane used in electrofiltration, where a conductive polymer layer serves as an electrode integrated with a porous support. Under applied potential, simultaneous filtration and electrochemical reactions occur, enabling in situ degradation of contaminants while maintaining permeate flux. Reproduced from Ref. [28] with permission from MDPI.

Electrically conductive polymers provide a third paradigm, where the membrane itself participates in an electrochemical process under applied voltage. These conductive polymer membranes (often polyaniline, polypyrrole, PEDOT: PSS blends) are used as either the anode or cathode surface while filtration is occurring (Figure 2). In effect, the membrane supports both separation and in situ electrolysis, as water is pushed through, contaminant molecules come into contact with the biased conductive network, which can oxidize or reduce them. For example, a positively biased anode made of polyaniline-coated polyimide can generate reactive oxygen species (like HO^•^, H_2_O_2_) at its surface, attacking organics as they pass through the pores. On the cathode side, the conductive polymer can reduce oxyanions or break down chlorinated compounds via electron transfer.

These systems blur the line between membrane and electrode. The polymer’s inherent conductivity allows charge carriers to migrate across the membrane thickness, so pollutant oxidation can occur throughout the filter bed rather than only on the extreme surface. Recent reports demonstrate that polyaniline/polyimide composite membranes, when connected to a few volts of bias, achieve near-quantitative removal of dye molecules with minimal energy use, because the oxidizing polymer regenerates over time. (The very act of filtration enhances mass transport, so oxidation is not confined to a stagnant layer.) From an engineering perspective, conductive polymer membranes are interesting because they require no separate reactor volume; the treatment happens concurrently with filtration. Characteristically, such membranes are tested under electrofiltration conditions: one measures pollutant removal with applied current vs. without. The presence of conductivity is often confirmed by a four-point probe on dry samples, and the magnitude of generated oxidants (measuring H_2_O_2_ or HO^•^ probes in the effluent) correlates with removal efficiency. Over time, it is important to monitor polymer oxidation (conductive polymers can degrade under high potential or long operation) and gas evolution (to avoid bubble-induced delamination). In summary, conductive polymer membranes turn the polymer into a third electrode, providing reactive sites and electron flow paths that enhance degradation of trace pollutants during filtration.

### 2.4. Porous Conjugated Polymer Membranes

**Figure 3:**
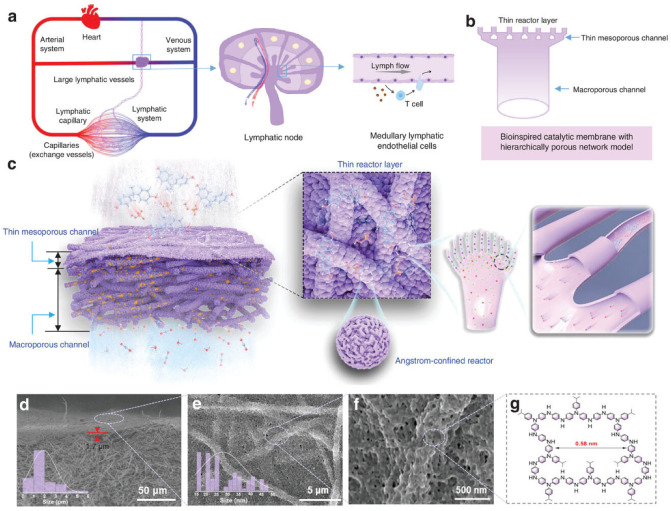
Biological inspiration and schematic of bioinspired catalytic membrane for micropollutants’ removal. (**a**) Hierarchical networks of blood and lymphatic vessels and tissue immunosurveillance of thin lymphatic endothelial cells. (**b**) Inspired hierarchically porous network model for a catalytic membrane. (**c**) Schematic illustration of the bioinspired catalytic membrane built on PMS-based AOP. It contains a thin layer of micropores for micropollutants’ degradation on the top, and branched mesopores and micropores for increasing permeability. (**d**–**f**) SEM images of the as-prepared bioinspired catalytic membrane containing macropores and mesopores under different magnifications. (**g**) Proposed chemical structure of PTPA with intrinsic micropores. Reproduced from Ref. [16] with permission from Wiley.

Another category involves porous conjugated polymer membranes, such as metal–organic frameworks (MOFs), covalent organic frameworks (COFs), conjugated microporous polymers (CMPs), or linear conjugated polymers cast into films [29]. These materials combine nanoscale porosity with π-conjugated backbones, creating a confined reactor environment. When used in membranes, they can enforce very short residence distances and strong interactions between trapped oxidants (Fenton reagents, peroxides) and substrates, often favoring non-radical pathways [30]. For example, in an oxygen-rich flow, an azo- or imine-linked polymer pore will preferentially transfer energy to molecular oxygen, generating singlet oxygen (^1^O_2_) as the dominant reactive species. This can be advantageous because ^1^O_2_ is selective and less susceptible to quenching by common water constituents than hydroxyl radicals [31]. In one demonstrated case, a nitrogen-rich porous polymer membrane generated ~98–99% singlet oxygen when activating persulfate, leading to efficient dye degradation at high flux [32]. The design rule here is that the polymer’s pore structure and conjugation control the chemistry. Small pores (angstrom-scale) can stabilize excited intermediates, as illustrated by the hierarchical porous architecture of such membranes (Figure 3) [16].

These membranes are characterized by measuring species like ^1^O_2_ (using sensor probes) and HO^•^ (using scavenger assays) under operating conditions. Unlike free radical polymer membranes, these conjugated polymers typically do not have unpaired electrons at rest, but their excited or charged states under bias or illumination lead to reactive oxygen generation [33]. Over time, pore fouling and photo-oxidative degradation of the polymer itself require careful monitoring. Stability tests often monitor retention of porosity (BET surface area) and photocatalytic efficiency after extended use.

In summary, porous conjugated polymer membranes act as confined photocatalysts or electrocatalysts, whereby the polymer’s intrinsic light/charge absorption and rigid porous network guide which reactive oxygen species are formed, enabling high-rate oxidation of micropollutants under flow-through conditions [34]. Together, the four aforementioned polymer classes—radical-rich networks, redox-active systems, conductive polymers, and porous conjugated frameworks—define the material lexicon of intrinsic reactive polymer membranes. Each class offers different tradeoffs in terms of mechanism, selectivity, and durability. In real designs, elements of multiple classes may be combined (for instance, a conductive polymer that also contains redox-active pendants, or a conjugated porous film doped with stable radicals) [35]. This review will adopt this taxonomy to systematically explore how polymer chemistry, microstructure, and module integration affect the performance of reactive filtration systems in treating complex wastewaters.

Overall, these membrane classes differ not only in their reactive chemistry but also in their transport behavior, stability risks, and readiness for scale-up. Table 1 summarizes the main features of stable-radical, redox-active, conductive, and porous conjugated polymer membranes, comparing their reactive mechanisms, advantages, limitations, and scalability. This comparison highlights that no single class is universally superior; rather, the most suitable membrane design depends on the target contaminant, oxidant chemistry, operating mode, and required long-term stability.

## 3. Mechanistic Core: ROS Pathways and Membrane Microenvironments

### 3.1. Radical vs. Nonradical Oxidation

When polymers generate reactive oxygen species (ROS) in situ, the chemistry can be radical-dominated or nonradical (selective). Radical pathways (via HO^•^, SO_4_^•−^, or organic peroxyl radicals) involve chain reactions where a single initiator can produce many radical events. These are powerful but often unselective and easily quenched by background organics. Nonradical pathways (such as singlet oxygen ^1^O_2_ or surface-bound oxidants) tend to be more selective and can achieve similar degradation with much lower oxidant use. For example, recent work has shown that systems favoring ^1^O_2_ generation can degrade contaminants using less oxidant than comparable radical systems [36]. In practice, a polymer membrane that promotes radical chain chemistry will generate a broad spectrum of intermediates and often requires excess oxidant to sustain the chain. In contrast, a membrane microenvironment that produces ^1^O_2_ (or high-valent metal-oxo species) will steer oxidation through well-defined electronic excited states, limiting the formation of highly oxidizing radicals. This usually results in fewer toxic byproducts. Singlet-oxygen pathways allow deep contaminant oxidation with significantly lower peroxide doses, due to the lower activation barrier and higher utilization efficiency [37].

**Figure 4:**
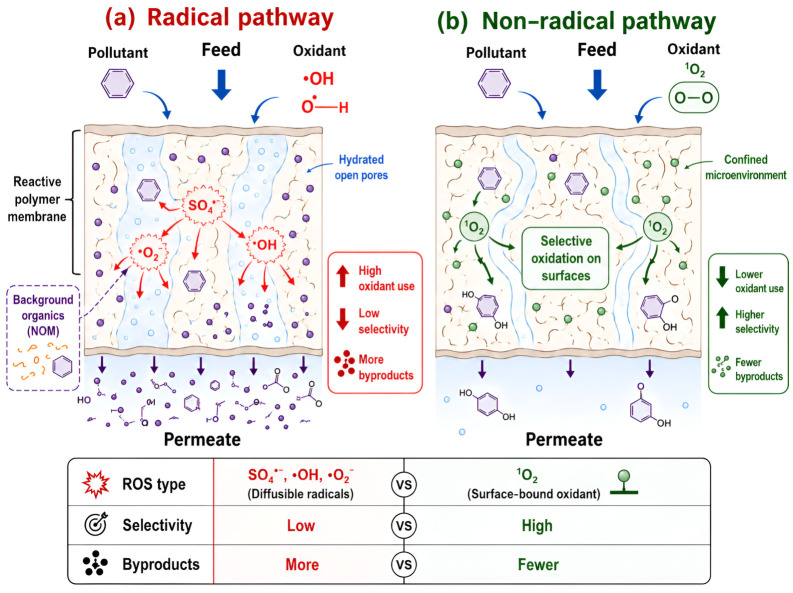
Schematic illustration of radical and non-radical oxidation pathways in flow-through reactive polymer membranes.

To clarify these mechanistic differences, Figure 4 schematically illustrates radical and non-radical oxidation pathways under flow-through membrane conditions. In radical-dominated systems, oxidants are activated to produce highly reactive species such as HO^•^ and SO_4_^•−^, which can rapidly attack pollutants but may also react with background organic matter. In non-radical systems, pathways such as ^1^O_2_ formation or surface-bound oxidation can provide more selective transformation within confined polymer environments.

**Figure 5:**
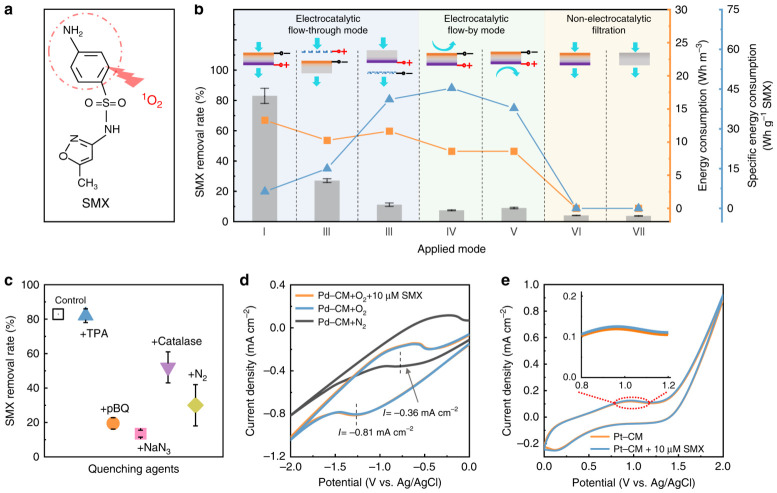
(**a**) Schematic illustrating the reaction between ^1^O_2_ and SMX. (**b**) SMX removal efficiency and electric energy consumption by Pd–Pt–CM under different electrocatalytic and conventional filtration modes. Modes I–III are for flow-through electrofiltration. For mode, I, the Pd–CM and Pt–CM surfaces of the Pd–Pt–CM serve as cathode and anode, respectively. Modes II and III apply each side of the Pd–Pt–CM as the cathode (mode II) or anode (mode III) and a Ti mesh plate as the counter electrode inserted in the feed chamber (mode II) or permeate chamber (mode III). Modes IV and V are electrocatalytic flow-by modes using the Pd- and Pt-functionalized surfaces as the cathode and anode, respectively. The reaction times of modes IV and V are the same as the filtration duration in mode I (i.e., 38 min). Modes VI and VII are conventional filtration modes using Pd–Pt–CM and CM without applying voltage. For each filtration mode, the feed solution contains 10 μM SMX and 100 mM Na_2_SO_4_. Error bars represent the standard deviation from triplicate experiments. In the schematic above each bar, the orange layer and purple layer represent Pd–CM and Pt–CM surfaces, respectively, and the blue arrows denote the direction of water flow. (**c**) ROS quenching by applying 10 mM TPA, 10 mM p-benzoquinone (pBQ), 2 mg L^−1^ catalase, and 10 mM sodium azide (NaN_3_) as quenchers for HO^•^, O_2_^•−^, H_2_O_2_, and ^1^O_2_, respectively. Measurements were performed in mode I with the respective quenching agent in the feed solution. Error bars represent the standard deviation from triplicate experiments. (**d**) CV curves of the Pd–CM surface in 100 mM Na_2_SO_4_ electrolyte at a scan rate of 0.1 V s^−1^. (**e**) CV curves of the Pt–CM surface in 100 mM Na_2_SO_4_ electrolyte at a scan rate of 0.1 V s^−1^. Reproduced from Ref. [15] with permission from Nature.

Under confinement (such as in nanoporous polymer matrices), pathway selectivity can shift. Narrow pores and hydrophobic pockets can stabilize excited states of oxygen, boosting the ^1^O_2_ yield, while suppressing free HO^•^ formation. Conversely, a hydrated, open polymer network tends to support conventional radical chemistry. In sum, whether a polymer membrane favors radical versus nonradical oxidation profoundly affects its performance and byproduct profile. A radical-rich system may completely mineralize organics but risks generating toxic intermediates (aldehydes, carboxylic acids, or increased bromate when bromide is present). A ^1^O_2_ dominant system often achieves cleaner transformations, as supported by recent studies combining quenching experiments and electrocatalytic filtration performance (Figure 5) [15]. Thus, understanding and controlling this selectivity is key to designing membranes that combine high conversion with environmental safety.

### 3.2. Polymer Microenvironment Descriptors

**Figure 6:**
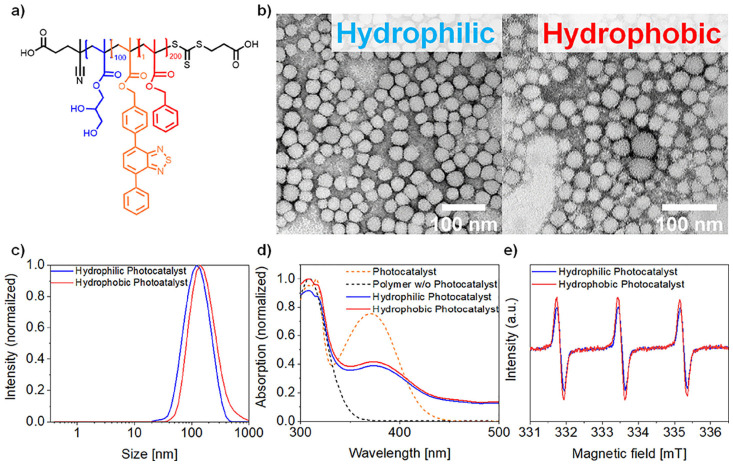
Characterization of the synthesized photocatalytic nanoparticles. (**a**) Chemical structure of the designed block copolymer. (**b**) Transmission electron microscopy (TEM) images of the hydrophilic and hydrophobic nanoparticles. (**c**) Dynamic light scattering (DLS) spectra for both nanoparticle systems in H_2_O. (**d**) UV/VIS-absorption spectrum of both nanoparticles, showing similar intensities at 373 nm in DMSO. (**e**) Electron paramagnetic resonance spectra of both nanoparticle systems after 10 min of 465 nm LED irradiation in H_2_O with TEMPO trapping. Reproduced from Ref. [39] with permission from ACS.

The local environment around catalytic centers in a polymer membrane, its polymer microenvironment, strongly dictates which ROS are formed and how contaminants interact. Hydrophilic polymers imbibe more water, which can favor the formation and mobility of HO^•^ and other polar radicals. Hydrophobic matrices exclude water and concentrate nonpolar pollutants, often enhancing energy transfer to dissolved O_2_ (boosting ^1^O_2_). For instance, an amine-rich polymer membrane might hydrate and support Fenton-like hydroxyl generation, whereas a fluorinated aromatic polymer could favor superoxide or singlet pathways. Polymers bearing ionic groups (–COO^−^, –NH_3_^+^, sulfonate) create electrical microdomains. These attract counterions and repel like-charges, altering local pH and partitioning [38]. A cationic membrane containing protonated amine groups (–NH_3_^+^) can enrich anionic contaminants, such as nitrate and anionic PFAS species including PFOA and PFOS, while also attracting peroxydisulfate anions, potentially enhancing radical formation near the membrane surface. Conversely, an anionic membrane may concentrate metal cations (enhancing metal-catalyzed cycles) or exclude anions, which changes which precursors get activated. Charge density also affects water structure: higher charge typically means more bound water and slower diffusion of ROS. Conjugated or aromatic units in the polymer can donate or accept electrons, participating in electron-transfer chemistry. Polymers with strong electron donors (phenolic, amine groups) can quench radicals or easily reduce oxidants, while strong acceptors (nitro, imine) may trap electrons and stabilize radical intermediates. These motifs essentially tune the redox potential landscape. For example, a polymer rich in quinone moieties was found to preferentially cycle H_2_O_2_ via one-electron transfers, whereas a polymer with multiple phenyl-rich domains can favor nonradical pathways such as singlet oxygen generation, as supported by spectroscopic and EPR (electron paramagnetic resonance) characterization of hydrophilic and hydrophobic polymer systems (Figure 6) [39].

The affinity of a polymer for a given contaminant affects how long the molecule resides near catalytic sites. Hydrophobic pockets in a polymer membrane will pull in nonpolar pesticides and pharmaceuticals, raising their local concentration and reaction rate. Meanwhile, water-soluble ROS generated in that microenvironment can more readily attack these enriched organics. Partition coefficients between polymer and water thus modulate effective kinetics. In practice, engineers measure contact angles or octanol-water partitioning of probe molecules to estimate how different contaminants will concentrate in the membrane [40]. For instance, a highly charged hydrophilic polymer might swell, increasing free volume (benefiting transport but diluting charge), while introducing hydrophobic domains can create nano-phase separation (like amphiphilic block copolymers). Advanced characterization based on swelling studies, zeta potential, and porosimetry can quantify these factors. Conceptually, it is possible to view each polymer pore as a tiny reaction vessel: its water content, electric field, and electronic structure determine which ROS are most stable inside it and which substrates are co-localized [41]. Altogether, tailoring these microenvironment properties allows control of ROS yield and selectivity in real matrices. For example, one might design a membrane with alternating hydrophilic/hydrophobic domains to first adsorb a and then bring it into contact with a hydrophilic catalytic zone that generates HO^•^ for oxidation [42].

### 3.3. Mechanism Testing and Verification

Rigorous verification of the ROS mechanism in flow-through membranes is essential, especially to avoid false positives. Recent guidance emphasizes multi-pronged detection strategies. Use EPR spin-trapping to detect short-lived radicals: for instance, DMPO can trap HO^•^ or SO_4_^•−^ (forming characteristic spectra), while TEMP or tempone can trap singlet oxygen. Complement this with chemical probes. terephthalic acid or coumarin for HO^•^ (which becomes fluorescent upon reaction), furfuryl alcohol for ^1^O_2_, or iodide for H_2_O_2_. Always validate that each probe or trap is selective and not reacting with unintended species (DMPO can also oxidize to DMPOX in some systems). Run the membrane system with and without membrane, with and without added oxidant, and with known quenchers. For example, if excess sodium azide (a ^1^O_2_ quencher) is used without checking that it does not also scavenge H_2_O_2_ or HO^•^, conclusions will be misleading [43].

The reported steady-state concentrations of reactive species in AOPs are typically extremely low (10^−16^–10^−8^ M). This means analytical sensitivity is crucial for accurately detecting and quantifying transient ROS, distinguishing the dominant oxidation pathway, and avoiding misinterpretation of scavenger or probe-based experiments. EPR should be done with sufficient signal averaging and under conditions similar to real operation (flow rate, membrane area). Probe tests should be performed in situ (adding the probe to the feed or effluent in a side-by-side experiment) and quantified by HPLC or fluorescence. Steady-state assumptions used in batch tests may not hold under filtration, so it is best to measure profiles along the membrane. If the signal is barely above noise, comparisons (with membrane vs. without membrane) are more reliable than absolute quantification. A common pitfall is assuming that adding a scavenger turns off one ROS while leaving others unaffected. In reality, overlapping reactivity often occurs. For example, methanol can quench HO^•^ but also affects peroxyl radicals, while L-histidine (a common ^1^O_2_ quencher) can also react with HO^•^. Thus, one should use multiple scavengers in parallel and interpret trends rather than absolute stops. Ideally, use kinetic modeling, vary scavenger concentration and fit the degradation kinetics to a model including all plausible ROS, to extract contributions [44].

Real wastewater contains background substances (humics, nitrate, bicarbonate) that can produce or quench ROS themselves. When testing membranes on spiked clean water, also test spiked real water. For example, bicarbonate can convert HO^•^ into less-reactive carbonate radicals, while nitrite can produce NO_2_^•^ radicals. Such species can confound spin-trapping signals or mimic certain spectral features. Best practice is to measure a matrix blank (feed without the target pollutant) to characterize background ROS levels, and to run isotope-labeled (or surrogate) tracers to ensure that detected oxidation products truly come from the target. Every study should report operating pH, ionic strength, and presence of known ROS scavengers (like Cl^−^, CO_3_^2−^) in the feed, because these determine which ROS are likely present. Recent critical assessments of ROS probe methods emphasize that probe applicability and reaction rate constants should be evaluated under the specific experimental conditions to avoid misleading mechanistic assignments [43]. Finally, control experiments with inactive membranes (no reactive groups) under identical conditions are crucial to show that any observed degradation indeed requires the functional polymer [45].

Based on these considerations, ROS verification in flow-through reactive membranes should follow a minimum standardized protocol. First, probe and scavenger selection should be justified according to the expected ROS, oxidant chemistry, pH, and water matrix, and relevant second-order reaction rate constants should be reported or cited. Second, the ROS assignment should not rely on a single scavenger experiment, but should combine at least two complementary lines of evidence, such as EPR/spin-trapping, selective probe assays, quencher tests using typical scavengers (e.g., tert-butanol or isopropanol for HO^•^, methanol or ethanol for HO^•^/SO_4_^•−^, p-benzoquinone for O_2_^•−^, sodium azide, L-histidine, or furfuryl alcohol for ^1^O_2_, and catalase for H_2_O_2_), transformation-product analysis, or isotope/surrogate experiments [43,46,47]. Third, the same diagnostic tests should be performed under filtration conditions whenever possible, because residence time, local concentration polarization, and membrane partitioning can change the apparent ROS pathway compared with batch experiments. Finally, matrix blanks, oxidant-only controls, membrane-only controls, and inactive-membrane controls should be included to separate polymer-driven ROS generation from bulk oxidant reactions and background matrix effects.

By following these guidelines using multiple orthogonal detection methods, carefully chosen scavengers, and appropriate controls, researchers can confidently assign which ROS are active. This mechanistic rigor is particularly important in novel polymeric systems, where unanticipated redox processes (polymer self-oxidation or metal leaching) could otherwise be mistaken for true catalytic activity. Converging evidence from spectroscopy, probe chemistry, and quantification ensures that claims about “intrinsic radical versus singlet oxygen pathways are scientifically sound.

## 4. Framework for Oxidative Filtration Kinetics

Oxidative filtration differs from conventional membrane separation because pollutant removal is controlled by the simultaneous action of permeation, diffusion, adsorption, and chemical reaction. In a conventional pressure-driven membrane, the main performance indicators are typically flux, rejection, and fouling resistance [48,49]. In reactive polymer membranes, however, contaminants are not only transported or rejected; they may also become enriched near reactive domains, diffuse into the pore structure, and undergo transformation at catalytic or redox-active sites embedded within the membrane [12]. As a result, the observed removal cannot be interpreted solely in terms of apparent rejection, and a kinetic framework is needed to distinguish the respective contributions of transport, adsorption, and true chemical degradation.

**Figure 7:**
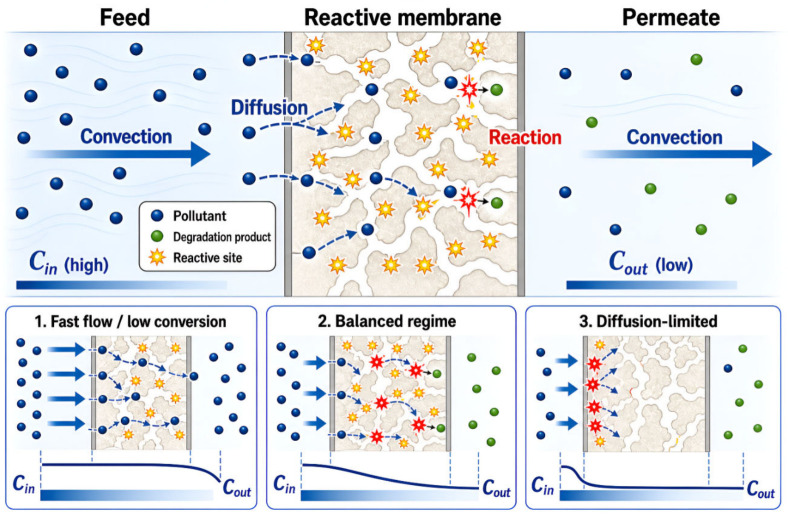
Schematic of reactive transport during oxidative filtration. The top panel shows coupled convection, diffusion, and reaction across the membrane, while the bottom panels illustrate three regimes: fast flow (low conversion), balanced transport-reaction, and diffusion-limited behavior.

During oxidative filtration, water first passes through an external hydrodynamic boundary layer, where concentration polarization may increase the local concentrations of pollutants and oxidants near the membrane surface. After entering the membrane, convective transport through the pores is coupled with intrapore diffusion and reaction [50]. Depending on the membrane architecture and the distribution of active sites, the dominant reaction zone may be located near the surface, within the pore network, or across the full thickness of the reactive layer. In this sense, oxidative filtration resembles a flow-through catalytic reactor, with the important distinction that the catalyst is integrated directly into the membrane structure and the reacting solution is forced through the reactive medium. Figure 7 schematically illustrates this coupled transport-reaction behavior during membrane passage and highlights three representative operating regimes: fast flow with low conversion, a balanced transport-reaction regime, and a diffusion-limited regime. These regimes can be interpreted using dimensionless transport–reaction parameters, particularly the Damköhler number, which relates reaction rate to convective residence time, and the Péclet number, which compares convective and diffusive transport. These parameters provide a quantitative basis for distinguishing flow-limited, reaction-balanced, and diffusion-influenced operation and are discussed in detail in Section 4.2.

### 4.1. Reactive Permeation Framework

In reactive polymer membranes, apparent pollutant removal can result from several overlapping mechanisms rather than a single pathway. Depending on membrane structure, pollutant properties, and operating conditions, removal may involve size exclusion, electrostatic rejection, adsorption, or partitioning into the polymer phase, catalytic oxidation, electrochemical transformation, or retention/passage of transformation products. This distinction is important because a decrease in parent-compound concentration does not necessarily confirm complete degradation. For example, a pollutant may be temporarily adsorbed inside the membrane, rejected by charge or size effects, or converted into smaller products that pass into the permeate. Therefore, mechanistic interpretation requires control experiments and mass-balance analysis, including non-reactive membranes, oxidant-free operation, adsorption tests, retentate/permeate analysis, and transformation-product identification. Such separation of rejection, adsorption, and degradation is essential before kinetic parameters are assigned to true chemical transformation.

A useful starting point for describing oxidative filtration is a one-dimensional steady-state convection–diffusion–reaction model across the membrane thickness. For a pollutant concentration C moving along the membrane coordinate z, the local balance can be written as [51,52]:

(1) $$v\frac{dC}{dz}=D_{eff}\frac{d^{2}C}{dz^{2}}-k_{rxn}C$$

where v (m/s) is the interstitial velocity through the membrane pores, C (mol/m^3^) is the pollutant concentration, z (m) is the coordinate across the reactive layer, D_eff_ (m^2^/s) is the effective diffusivity inside the membrane, and k_rxn_ (s^−1^) is the apparent reaction rate coefficient. The left side of Equation (1) represents convective transport caused by permeation, while the first term on the right side represents diffusion inside the membrane pores. The final term describes pollutant removal by chemical reaction.

Equation (1) is intentionally simple, but it captures the central feature of oxidative filtration: reaction and transport occur at the same time. If the reaction term is removed, the model describes non-reactive permeation and diffusion. If the convective term is small, the system approaches a diffusion–reaction problem. If diffusion is fast relative to reaction, the system behaves closer to plug flow conversion through a reactive layer. Thus, Equation (1) provides a common framework for interpreting different membrane configurations, including porous catalytic layers, conductive polymer membranes, and redox-active polymer films.

For a first-order reaction, the reaction rate can be expressed as Equation (2) [53].

(2) $$r_{rxn}=k_{rxn}C$$

where r_rxn_ (mol s^−1^ m^−3^) is the local reaction rate. This first-order form is often used when the oxidant is present in large excess or when its concentration remains nearly constant during the experiment. In systems where both pollutant and oxidant concentrations vary significantly, a second-order (Equation (3)) or Langmuir-type expression (Equation (4)) may be more appropriate [54].

(3) $$r_{rxn}=k_{app}C_{P}C_{O}$$

where C_P_ and C_O_ represent pollutant and oxidant concentrations, respectively, and k_app_ is an apparent second-order rate coefficient (m^3^ mol^−1^ s^−1^). When adsorption strongly controls local concentration, the Langmuir equation is used:

(4) $$r_{rxn}=\frac{k_{s}K_{ads}C}{1+K_{ads}C}$$

Here, k_s_ (mol m^−3^ s^−1^) is a surface reaction coefficient, and K_ads_ (m^3^ mol^−1^) is an adsorption equilibrium coefficient. Equation (4) is useful when pollutant enrichment near reactive sites is important, as in hydrophobic, charged, or porous polymer domains.

A key difference between oxidative filtration and conventional batch oxidation is the short contact time. In membrane systems, the residence time within the reactive layer may range from milliseconds to seconds, depending on flux, layer thickness, and porosity [55]. This can be estimated as Equation (5).

(5) $$\tau =\frac{L_{eff}}{v}$$

where τ (s) is the residence time, and L_eff_ (m) is the effective thickness of the reactive layer. Equation (5) highlights why “milliseconds matter” in oxidative filtration. If water passes through the active layer too quickly, even a highly reactive material may show limited single-pass conversion. Therefore, the membrane must provide either very fast reaction kinetics, strong local enrichment of pollutants, efficient oxidant activation, or an optimized flow path that increases effective contact time without causing excessive pressure drop.

The conversion of a pollutant under ideal plug flow conditions can be approximated as:

(6) $$X=1-\exp(-k_{rxn}\tau )$$

where X is the fractional conversion. This expression shows that conversion depends on the product of the rate coefficient and residence time. When k_rxn_ × τ is small, conversion is low; when k_rxn_ × τ is large, near-complete conversion can be achieved. This relationship explains why thin catalytic layers with high activity are attractive. They reduce hydraulic resistance while maintaining sufficient reaction within a short residence time [15].

However, real oxidative membranes rarely behave as ideal plug flow reactors. Flow distribution may be non-uniform, pores may have different residence times, and fouling can shift the location of the reaction zone. As a result, residence time distribution becomes important. A membrane with the same average residence time can show different conversion depending on whether the flow is uniformly distributed or dominated by channeling. For this reason, reactive permeation models must eventually include both average kinetics and hydrodynamic heterogeneity.

### 4.2. Dimensionless Mapping for Cross-Study Comparison

One major challenge in comparing oxidative filtration studies is that experiments are performed under different membrane thicknesses, fluxes, pore structures, pollutant concentrations, oxidant doses, and water matrices. Apparent rate constants alone are therefore insufficient for meaningful comparison. Dimensionless numbers provide a more general way to classify the operating regime and compare systems across different scales.

The most important dimensionless number for reactive filtration is the Damköhler number. For a permeating membrane system, a permeation Damköhler number can be defined as Equation (7).

(7) $$Da_{perm}=\frac{k_{rxn}L_{eff}}{v}$$

where Da_perm_ compares the characteristic reaction rate with the convective transport rate through the reactive layer. When Da_perm_ << 1, the pollutant passes through the membrane faster than it reacts, and single-pass conversion is low. When Da_perm_ >> 1, the reaction is fast relative to permeation, and high conversion can occur within one pass. Thus, Da_perm_ provides a direct link between material activity, membrane thickness, and operating flux [56].

Typical values of Da_perm_ can be interpreted using the first-order plug flow relation as Equation (8).

(8) $$Da_{perm}=k_{rxn}\tau =-\ln(1-X)$$

In general, Da_perm_ < 0.1 corresponds to low single-pass conversion, Da_perm_ ≈ 1 indicates a transition region where reaction and permeation occur on comparable time scales, and Da_perm_ > 3 corresponds to high conversion (>95%) under ideal first-order plug flow conditions [57]. For example, Zhao et al. reported 82.9% sulfamethoxazole removal in a Janus electrocatalytic flow-through membrane with a membrane residence time of approximately 23 s, corresponding to an estimated Da_perm_ ≈ 1.77 using the first-order approximation [15]. Peng et al. also reported nearly complete persulfate activation in a Ti_4_O_7_-reactive electrochemical membrane with a residence time below 10 s, indicating operation in a high-Da regime where reaction is fast relative to permeation [55].

Diffusion limitations inside pores can be described using a Thiele-type modulus in Equation (9) [58].

(9) $$\Phi =L_{c}\sqrt{\frac{k_{rxn}}{D_{eff}}}$$

where Φ is the Thiele modulus, and L_c_ (m) is a characteristic diffusion length, such as pore radius, active-layer thickness, or catalyst-domain size. When Φ << 1, diffusion is fast relative to reaction, and most active sites are accessible. When Φ >> 1, reaction is fast, but diffusion is limiting, meaning that only part of the reactive domain is effectively used. In porous polymer membranes, this distinction is important because increasing active-site density does not always improve performance if pollutants or oxidants cannot reach those sites efficiently [59].

The relative importance of convection and diffusion can be expressed using the Péclet number as shown in Equation (10) [60].

(10) $$Pe=\frac{L_{eff}v}{D_{eff}}$$

Pe compares convective transport to diffusive transport. A high Pe indicates that flow dominates transport, while a low Pe indicates that diffusion plays a larger role. In oxidative filtration, high Pe values can increase throughput but may reduce contact time. Low Pe values may improve reaction opportunity, but they can reduce the efficiency and increase the risk of concentration polarization. Therefore, an optimized system must balance throughput and reaction efficiency [61].

These dimensionless groups allow membrane performance to be mapped into different regimes. For example, a system with low Da_perm_ and high Pe is primarily limited by insufficient contact time. In this case, increasing active-layer thickness, reducing flux, or improving catalytic activity may increase conversion. A system with high Da_perm_ but high Φ is diffusion-limited; in this case, adding more active sites may have little benefit unless pore accessibility is improved. A system with high Da_perm_ and low φ is reaction-efficient and transport-accessible, representing a desirable operating window.

In practical membrane operation, these regimes can be linked directly to observable performance trends. For example, a membrane operating at high flux but showing low removal is likely in a low-Da_perm_/high-Pe regime, where convection is strong but the contact time is insufficient for complete degradation. Reducing flux, increasing active-layer thickness, or improving intrinsic reaction rate would be expected to increase conversion in this case. Conversely, if high removal is achieved only at very low flux, the system may rely mainly on long residence time rather than high catalytic activity, which limits productivity. A high Φ value provides another warning: even if the apparent reaction rate is high, pollutants and oxidants may react mainly near the outer pore region, leaving internal active sites underused. Therefore, simultaneous reporting of removal efficiency, flux or permeance, Da_perm_, Pe, and Φ can help distinguish whether membrane performance is controlled by residence time, convective throughput, intrapore diffusion, or true reaction kinetics.

This interpretation also helps link results from different studies in a more critical way. Similar removal efficiencies do not necessarily indicate similar membrane performance, because they may arise from different combinations of residence time, active-layer thickness, adsorption, oxidant dose, and intrinsic reaction rate. For example, high removal obtained at very low flux may mainly reflect extended contact time, whereas comparable removal at higher flux suggests faster reaction kinetics or more efficient mass transfer. Likewise, increasing the number of reactive sites is useful only when Φ remains low enough for pollutants and oxidants to access those sites during permeation. Therefore, quantitative comparison should consider removal together with flux or permeance, oxidant utilization, residence time, Da_perm_, Pe, and Φ. This systems-level reading makes it easier to identify whether reported improvements come from better polymer chemistry, improved transport architecture, or simply more favorable operating conditions.

Fouling complicates this analysis because it changes both transport and reaction conditions. Fouling can reduce pore size, lower effective diffusivity, increase hydraulic resistance, and shift the location of reaction zones [9]. These changes alter D_eff_, v, Pe, Φ, and Da_perm_ at the same time. For example, if fouling slows permeation, Da_perm_ may increase because the residence time becomes longer. This can lead to an apparent increase in pollutant conversion, even though the membrane is becoming hydraulically less efficient. Conversely, if fouling blocks reactive sites or scavenges reactive oxygen species, conversion may decrease even when residence time increases. Therefore, a change in removal efficiency during long-term operation cannot be interpreted without also monitoring flux, pressure, oxidant consumption, and membrane condition [62].

This dimensionless approach is useful because it prevents misleading comparisons. Two membranes may show the same pollutant removal, but one may operate at low flux with long residence time, while the other operates at high flux with fast reaction kinetics. Similarly, a high apparent conversion may reflect strong adsorption or long contact time rather than superior catalytic activity. Reporting Da_perm_, Φ, and Pe alongside removal efficiency would make cross-study comparison more meaningful and would help identify whether improvements originate from chemistry, transport, or operating conditions [60].

### 4.3. Kinetic Modeling Strategy

Kinetic modeling of oxidative filtration can be viewed as an inverse problem, where experimental permeation data are used to estimate reaction and transport parameters. However, this requires careful separation of adsorption, physical rejection, diffusion, and true degradation. Without this separation, an apparent rate constant may include multiple effects and cannot be compared across studies.

The first step is to quantify adsorption and partitioning. Many pollutants can accumulate within polymeric membranes due to hydrophobic interactions, electrostatic attraction, hydrogen bonding, or affinity toward catalytic domains. This enrichment increases the local concentration near reactive sites and may enhance degradation rates. However, adsorption alone can also appear as removal if only feed and permeate concentrations are measured. Therefore, adsorption tests without oxidant or without active reaction are needed to determine the local enrichment factor or adsorption equilibrium constant [63].

A simple relationship between membrane-phase concentration and aqueous concentration can be described by Equation (11), following the general concept of pollutant partitioning and sorption into membrane polymers [63].

(11) $$C_{m}=K_{p}C_{w}$$

where C_m_ (mol/L_membrane_) is the pollutant concentration in the membrane phase, C_w_ (mol/L_water_) is the pollutant concentration in water, and K_p_ is the polymer-water partition coefficient (dimensionless). When K_p_ > 1, the pollutant is enriched in the membrane, and the local reaction rate may be higher than expected from bulk concentration alone. For reactive membranes, kinetic models should therefore use the local concentration at the reaction zone rather than only the feed concentration [63].

The second step is to measure baseline permeation in the absence of reaction. This can be done by operating the membrane without oxidant, without applied potential, or with deactivated catalytic sites. Breakthrough curves at different flow rates can then be used to estimate effective diffusivity, dispersion, adsorption capacity, and possible physical rejection. A non-reactive mass balance provides the baseline against which reactive removal is evaluated.

The third step is to conduct reactive experiments under controlled variations in flow rate, oxidant concentration, and pollutant concentration. These experiments should include measurements of feed concentration, permeate concentration, oxidant residual, and transformation products where possible. Running the system at multiple fluxes is especially important because changing flux changes residence time within the active membrane or reactor zone [64]. If conversion decreases predictably with increasing flux, the system is likely governed by reaction–residence time coupling. If conversion remains unchanged over a wide flux range, the process may be limited by adsorption, physical rejection, or very fast surface reaction.

For a plug flow approximation, the apparent first-order rate coefficient can be estimated from conversion and residence time, as shown in Equation (12), which is derived from the standard first-order plug flow reactor expression [57].

(12) $$k_{app}=\frac{-\ln(1-X)}{\tau }$$

where k_app_ is the apparent first-order rate coefficient, X is pollutant conversion, and τ is residence time. Equation (12) is useful for first-pass comparison, but it must be used cautiously. It assumes uniform flow, first-order behavior, negligible axial dispersion, constant oxidant concentration, and no significant adsorption contribution. In many oxidative membranes, these assumptions are only partially valid [64].

A more complete model may include adsorption-enhanced reaction, as shown in Equation (13). This approach is consistent with the broader literature on micropollutant partitioning into membrane polymers and adsorption-assisted photocatalytic degradation [63].

(13) $$r_{rxn}=k_{rxn}K_{p}C_{w}$$

In this case, the observed reaction rate increases with both the intrinsic reaction coefficient and the enrichment factor. This expression helps distinguish two different design strategies: increasing intrinsic catalytic activity or increasing pollutant partitioning into the reactive layer. Both may improve removal, but they have different implications for selectivity, fouling, and byproduct formation.

For electrochemical or conductive polymer membranes, charge input must also be incorporated. A useful electrochemical exposure metric is charge per treated volume (Equation (14)), because applied voltage alone does not determine electrochemical dose; current, flow rate, conductivity, and reactor geometry strongly influence the actual charge delivered to the treated water [65].

(14) $$Q_{V}=\frac{\left(I\times t\right)}{V_{treated}}$$

In Equation (14), Q_v_ (C/m^3^) is the charge per treated volume, I (C/s) is current, t (s) is operating time, and V_treated_ (m^3^) is the treated water volume. In a continuous flow, it can be described as Equation (15).

(15) $$Q_{V}=\frac{I}{Q_{flow}}$$

where Q_flow_ (m^3^/s) is the volumetric flow rate. Reporting charge per volume is important because applied voltage alone does not determine electrochemical dose. Two systems operating at the same voltage may deliver very different current densities and charge inputs depending on membrane conductivity, electrode spacing, water conductivity, and flow rate.

For energy comparison, electrical energy per order can be used (Equation (16)). EEO is a standardized figure of merit introduced for advanced oxidation technologies to compare the electrical energy required to reduce contaminant concentration by one order of magnitude in a defined volume of water [66].

(16) $$EEO=\frac{E_{elec}}{V_{treated}\log(\frac{C_{in}}{C_{out}})}$$

EEO (kWh/m^3^) is the electrical energy required to reduce pollutant concentration by one order of magnitude, E_elec_ (kWh) is the consumed electrical energy, V_treated_ is the treated volume, C_in_ is influent concentration, and C_out_ is effluent concentration. This metric is particularly useful when comparing electrofiltration systems with UV-based or other electrically driven advanced oxidation processes [67].

For example, Zhao et al. reported a Janus electrocatalytic flow-through membrane for sulfamethoxazole removal, where the optimized flow-through mode achieved 82.9% SMX removal with an energy consumption of only 13.3 Wh/m^3^ of permeate, corresponding to 0.0133 kWh/m^3^ under the reported conditions [15]. This example illustrates the low energy demand that can be achieved when electrochemical reaction and membrane permeation are coupled in a confined flow-through architecture. In contrast, UV-based AOPs often show higher energy requirements; a broad assessment of high-energy oxidation–reduction processes reported EEO values ranging from 0.6 to >300 kWh m^−3^ order^−1^, depending on the process, contaminant, and water matrix. For dye decolorization, one study reported minimum EEO values of 1.68 kWh m^−3^ order^−1^ for O_3_/MnO_2_ under 365 nm irradiation and 2.01 kWh m^−3^ order^−1^ for UV/H_2_O_2_/Fe^3+^ under 254 nm irradiation [68,69]. These examples show why EEO is useful for comparing electrofiltration with UV- or ozone-based advanced oxidation processes, while also emphasizing that values depend strongly on pollutant type, reactor design, and water composition.

### 4.4. Parameter Identifiability and Reporting

A major limitation in many reactive membrane studies is that fitted rate coefficients are reported without uncertainty or without sufficient information to judge whether the parameter is identifiable. A parameter is identifiable when the available data contain enough information to estimate it uniquely. For example, if experiments are conducted at only one flow rate and one initial concentration, the data may not be sufficient to distinguish the individual effects of k_rxn_, D_eff_, and adsorption strength. Different parameter combinations may describe the outlet concentration equally well, leading to large confidence intervals or standard deviations. In such cases, the fitted rate constant may be statistically non-significant and should not be interpreted as a reliable or physically meaningful parameter.

Reliable kinetic extraction requires data across different operating regimes, since insufficient or poorly informative datasets can lead to practical non-identifiability of fitted kinetic parameters [70]. Moderate conversion values are often the most informative. If conversion is too low, analytical error dominates. If conversion is nearly complete, many rate constants can explain the result. Experiments that cover a range of residence times, ideally producing conversions from low to high values, provide stronger parameter constraints. Similarly, testing at different oxidant concentrations can help determine whether the reaction is pseudo-first-order or dependent on oxidant availability [71].

Uncertainty analysis should accompany fitted kinetic parameters [72]. Confidence intervals, sensitivity analysis, or bootstrap methods can be used to estimate how measurement errors affect the fitted values. At a minimum, studies should report uncertainty in flux, concentration, conversion, and fitted rate constants. For oxidative membranes, it is also important to report whether parameters were obtained under clean conditions, fouled conditions, or after regeneration, because fouling changes the effective transport and reaction environment.

To further quantify the reliability of the estimated kinetic parameters, the standard deviation (SD) of each fitted parameter can be calculated from the residual error and the sensitivity of the model response to that parameter (Equation (17)) [73,74]. In this approach, the SD of the ith parameter, σ_i_, is obtained from the residual sum of squares between the experimental and calculated values, corrected by the number of experimental observations (N) and the number of fitted parameters (P). The calculation also includes the ith diagonal element of the inverse sensitivity matrix, which reflects how strongly the model output depends on the corresponding fitted parameter.

(17) $$\sigma_{i}=\sqrt{\frac{m_{ii}^{-1}x^{2}}{N-P}}$$

The sensitivity matrix (m_ij_) is constructed from the partial derivatives of the model response with respect to each fitted parameter (Equation (18)). These derivatives can be evaluated numerically by applying a small perturbation (δ) to each parameter and calculating the resulting change in the model output. To ensure stable and accurate derivative estimation, the perturbation should be sufficiently small relative to the parameter value; in previous kinetic modeling studies, a perturbation of 10^−4^ × a_i_ has been used, where a_i_ is the estimated value of the ith parameter (Equation (19)) [75].

(18) $$m_{ij}=\sum_{n=1}^{N}\frac{\partial F_{n}}{\partial a_{i}}\times \frac{\partial F_{n}}{\partial a_{j}}$$

(19) $$\frac{\partial F_{n}}{\partial a_{i}}=\underset{\Delta a_{i}\to 0}{\lim}\frac{\Delta F_{n}}{\Delta a_{i}}\approx \frac{F_{n}(a_{i}+\delta )-F_{n}(a_{i})}{\delta }$$

Using this procedure, the standard deviation provides a practical measure of the uncertainty associated with each fitted parameter. A parameter may be considered statistically meaningful when its estimated value is larger than its corresponding standard deviation [74,76]. Therefore, calculating σ_i_ for all fitted parameters allows the model to distinguish well-supported parameter estimates from values that are poorly constrained by the available experimental data. This analysis is particularly useful in oxidative filtration models, where multiple processes, including adsorption, diffusion, permeation, and reaction, can influence the fitted kinetic constants.

For cross-study comparability, kinetic parameters should be reported together with the physical and operating conditions under which they were measured. These include membrane thickness, active-layer thickness, membrane area, flux or permeance, pressure, feed concentration, oxidant dose, pH, ionic strength, temperature, water matrix, and operation time. Reporting only a rate constant without these details limits interpretation. In addition, dimensionless groups such as Da_perm_, Pe, and φ should be provided where sufficient data are available.

Overall, kinetic modeling provides a way to move oxidative filtration research beyond simple removal percentages. By combining reactive transport equations, dimensionless analysis, adsorption correction, and uncertainty reporting, it becomes possible to identify whether a membrane performs well because of intrinsic catalytic activity, favorable pollutant enrichment, improved residence time, or transport effects. This distinction is essential for rational materials design, fair cross-study comparison, and eventual scale-up of intrinsic reactive polymer membranes.

## 5. Materials Design Rules as Structure–Property–Performance Maps

### 5.1. Radical-Polymer Design Levers

Radical polymer membranes represent a promising class of materials in which the preservation and accessibility of active radical sites are central to performance during filtration. The stability of these radicals is governed by both molecular design and macromolecular architecture. At the molecular level, radical stabilization is typically achieved through resonance delocalization and steric protection [22]. For instance, nitroxide radicals are well known for their stability due to the presence of bulky tert-butyl substituents and conjugated frameworks that delocalize the unpaired electron, thereby suppressing recombination and side reactions. Embedding such stabilized radical moieties into a rigid and conjugated polymer backbone further enhances their lifetime by limiting segmental motion and reducing the probability of bimolecular quenching [19].

Beyond molecular considerations, the structural design of the polymer network plays a decisive role. Cross-linked architectures and interpenetrating polymer networks (IPNs) are particularly effective in immobilizing radical species while preserving porosity. In IPN systems, one polymer phase can host the radical functionality while the second phase provides mechanical robustness and controls pore architecture. This dual-function design enables high radical loading without compromising permeability [20]. For example, co-crosslinked vinyl polymer systems have been shown to successfully anchor radical species while maintaining sufficient water transport pathways. A critical challenge in such systems is distinguishing between radical degradation and membrane fouling during operation. This can be addressed by incorporating sacrificial hydrogen donors or antioxidant additives into the polymer matrix, which preferentially react with transient oxidizing species and protect the active radicals. Simultaneously, antifouling strategies such as grafting hydrophilic or zwitterionic polymer brushes onto the membrane surface can reduce the adsorption of macromolecular foulants. Monitoring techniques such as EPR spectroscopy allow independent tracking of radical density, enabling differentiation between chemical decay and physical pore blockage [21].

### 5.2. Porous Catalytic Layer Design Levers

**Figure 8:**
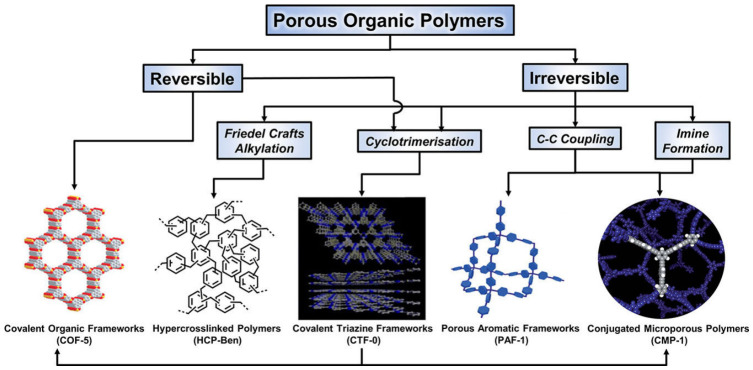
Types of porous organic polymer frameworks and their coupling chemistries. Porous polymers from left to right: covalent organic frameworks (COFs), hypercrosslinked polymers (HCPs), covalent triazine frameworks (CTFs), porous aromatic frameworks (PAFs), and conjugated microporous polymers (CMPs). Note that these classifications can overlap somewhat; for example, some COFs are also conjugated, and not all COFs reported are particularly crystalline. Reproduced from Ref. [78] with permission from ACS.

Porous catalytic layers, often constructed from conjugated polymer frameworks, serve as the functional core of oxidative membranes. In these systems, pore architecture is the dominant design parameter governing both catalytic efficiency and transport properties. Hierarchical porosity is typically employed to reconcile the competing requirements of high permeability and effective catalysis [77]. Micropores on the angstrom scale, commonly found in covalent organic frameworks (COFs) or conjugated microporous polymers, provide confined environments that enhance reactant catalyst interactions and promote the selective generation of reactive oxygen species such as singlet oxygen. This confinement effect not only increases reaction efficiency but also directs reaction pathways toward more selective oxidation processes. However, such small pores alone are insufficient to sustain practical water flux. Therefore, mesoporous and macroporous channels must be integrated into the membrane structure, often through the use of porous supports or templating strategies (Figure 8) [78].

This design requirement reflects a broader permeability–reactivity trade-off in highly functionalized porous polymer layers. Increasing the density of reactive groups, heteroatoms, or catalytic micropores can improve oxidant activation, pollutant enrichment, and ROS selectivity. However, excessive functionalization may reduce free volume, narrow pore channels, increase tortuosity, and limit water permeation. In such cases, additional active sites may not improve overall performance because pollutants and oxidants cannot access them efficiently during short membrane residence times. Therefore, porous catalytic layers should not be optimized only for maximum functional group density or surface area. Instead, effective designs require a balance between accessible reactive sites, connected transport pathways, thin active layers, and sufficient hydraulic permeability. This balance is especially important for oxidative filtration, where catalytic activity and mass transfer must simultaneously occur under flow-through conditions.

A widely adopted configuration involves the deposition of an ultrathin catalytic layer, typically on the order of tens to hundreds of nanometers, onto a highly porous support. This skin layer contains the active catalytic sites, while the underlying support provides mechanical stability and facilitates fluid transport [79]. Dual-layer or bilayer architectures have demonstrated exceptional performance in this regard, where a coarse support layer enhances permeability and a thin active layer ensures selectivity and fouling resistance. The chemical composition of the pore walls further modulates catalytic behavior. Incorporation of heteroatoms such as nitrogen, oxygen, or sulfur, as well as functional groups like imines or azo linkages, can tune the generation pathways of reactive oxygen species [80]. For example, nitrogen-rich frameworks have been shown to favor the production of singlet oxygen over hydroxyl radicals, thereby reducing the formation of undesirable byproducts. Additionally, selective uptake of target contaminants can be achieved by tailoring pore surface chemistry, such as introducing charged groups to attract oppositely charged species or modulating hydrophobicity to enhance interactions with organic pollutants. Given the short residence times in membrane systems, minimizing mass transfer limitations is essential [81]. This is typically achieved by reducing the thickness of the active layer and introducing nanostructured scaffolds or conductive additives that create interconnected transport pathways. Establishing quantitative relationships between structural parameters such as pore size distribution, layer thickness, and functional group density and performance metrics like flux, selectivity, and turnover frequency enables the construction of performance maps that guide the optimization of these catalytic systems [82].

### 5.3. Conductive/Electrocatalytic Membrane Design Levers

Conductive and electrocatalytic membranes integrate electron transport with separation processes, enabling electrochemically driven pollutant degradation. In these systems, the formation of efficient electron conduction pathways is the primary design consideration [83]. Pure conductive polymers such as polyaniline, polypyrrole, and poly(3,4-ethylenedioxythiophene) (PEDOT:PSS) often suffer from limited mechanical strength and insufficient porosity when used alone. Consequently, composite architectures are commonly employed [84]. Core–shell structures, such as carbon nanotube (CNT) cores coated with conductive polymers, provide a continuous electron transport network while maintaining surface redox activity. Similarly, interpenetrating polymer networks can combine conductive polymers with mechanically robust matrices, enabling simultaneous transport of electrons and ions while mitigating structural degradation under operating conditions [85].

The intrinsic conductivity of these materials is strongly influenced by the degree of π-conjugation and the level of chemical doping. Highly conjugated backbones, particularly those incorporating heteroaromatic units such as thiophene or pyrrole, facilitate efficient charge delocalization [86]. Doping with strong acids or incorporating conductive fillers such as graphene, carbon nanotubes, or metallic nanoparticles further enhances conductivity and electrocatalytic activity. Integration of the membrane into an electrochemical system requires careful attention to electrode assembly. Conductive membranes are typically fabricated directly onto current collectors, such as metal meshes, to minimize contact resistance and ensure uniform current distribution. Surface modification techniques and tailored flow-field geometries are often employed to prevent localized current hotspots and mitigate issues such as gas bubble accumulation during operation [45].

In evaluating the performance of electrocatalytic membranes, it is essential to adopt standardized metrics that enable meaningful comparison across systems. Charge-based metrics, such as the electrical charge passed per unit volume of treated water, provide a direct measure of electrochemical exposure analogous to fluence in photochemical systems [87]. Energy efficiency is commonly quantified using the Energy per Order (EEO), defined as the energy required to achieve a one-log reduction in contaminant concentration. Reporting additional parameters such as charge efficiency, electrode surface area, flow rate, and applied potential is critical for reproducibility and benchmarking [88]. The interplay between conductivity, permeability, and energy consumption defines a trade-off landscape in which highly conductive but dense membranes may achieve efficient oxidation at low voltage but suffer from limited flux, whereas more permeable structures may require high energetic input to reach comparable removal efficiencies. Mapping these relationships provides a framework for optimizing material composition and device architecture to meet specific performance targets in electrofiltration applications [89].

## 6. Fouling, Aging, and Safety

### 6.1. Fouling–Reactivity Coupling

The interplay between membrane fouling and chemical reactivity represents a critical aspect of reactive filtration systems operating under realistic wastewater conditions. In complex feed streams, foulants such as natural organic matter, proteins, oils, colloids, and biofilm components accumulate on membrane surfaces or within pores, thereby altering local transport and reaction environments. This accumulation can lead to the formation of a fouling layer that effectively concentrates both target pollutants and oxidants in confined regions. As a result, fouling can either enhance or suppress oxidative degradation depending on the chemical nature of the accumulated species. Certain foulants, including humic substances and phenolic compounds, act as radical scavengers and preferentially consume highly reactive species such as hydroxyl radicals, thereby reducing the fraction of oxidants available for target micropollutants [90]. In contrast, naturally occurring ions such as bromide and iodide may participate in oxidation pathways to form reactive halogen species, introducing alternative reaction routes and modifying selectivity. These competing interactions can shift the balance between selective and non-selective oxidation, often increasing the risk of undesirable byproduct formation when non-selective radicals dominate. In this sense, foulants act both as sinks for reactive species and as sacrificial substrates, fundamentally altering apparent reaction kinetics [91].

Conversely, reactive filtration processes can influence fouling behavior through in situ oxidation. Oxidative reactions at the membrane interface can degrade foulant layers, leading to partial self-cleaning. Macromolecular foulants may be fragmented into smaller, more hydrophilic species that detach more readily under shear forces, thereby maintaining higher permeate flux compared to conventional filtration. However, this benefit is accompanied by a significant trade-off, as reactive species may simultaneously attack the membrane material itself [25]. Prolonged exposure to oxidative conditions can accelerate polymer degradation, reduce mechanical stability, and diminish catalytic activity. As a result, system design must carefully balance fouling mitigation and material durability [92]. Strategies such as intermittent oxidation cycles or staged treatment configurations, where inert pre-filtration removes bulk foulants prior to reactive treatment, can mitigate these competing effects. Quantitative evaluation of this coupling is often achieved through fouling reactivity maps, in which flux and pollutant conversion are analyzed as functions of foulant concentration, enabling identification of optimal operating regimes [93].

### 6.2. Polymer Aging, Leaching, and Structure Drift

The long-term viability of reactive membranes depends on their ability to maintain both structural integrity and functional performance under prolonged chemical and mechanical stress. Chemical aging is primarily driven by oxidative degradation mechanisms, including chain scission and transformation of functional groups [94]. For example, widely used membrane polymers such as polyethersulfone and polyamide are vulnerable to attack by reactive oxygen species, particularly hydroxyl radicals, resulting in cleavage of sulfone or amide bonds. These changes manifest as the formation of new carbonyl-containing species and a reduction in molecular weight, which can be detected through spectroscopic techniques such as Fourier-transform infrared spectroscopy or nuclear magnetic resonance. In radical-based systems, electron paramagnetic resonance spectroscopy provides a direct measure of active site density, allowing the monitoring of radical decay over time [95].

Next to chemical aging, mechanical stability is equally important, as oxidative degradation can lead to embrittlement and loss of flexibility. Standard mechanical tests, including tensile strength and elongation at break, should be conducted before and after accelerated aging protocols to assess durability [19]. A material may be considered robust if its mechanical properties remain largely unchanged after prolonged exposure to oxidative environments. In parallel, catalyst leaching must be carefully evaluated, particularly in systems incorporating metal-based active sites or nanoparticles [96]. Analytical techniques such as inductively coupled plasma mass spectrometry or ultraviolet-visible spectroscopy can detect trace levels of leached species in the permeate. Ensuring that leaching remains below regulatory thresholds is essential for safe operation [97].

To comprehensively evaluate membrane durability, a combined performance index can be defined that integrates flux recovery, pollutant removal efficiency, and structural integrity. Flux recovery reflects the ability of the membrane to regain permeability after cleaning cycles, while retention of pollutant removal indicates sustained catalytic functionality [98]. Structural integrity can be assessed through a combination of spectroscopic and mechanical measurements. Long-term cyclic testing under realistic conditions, including repeated fouling and cleaning cycles, provides insight into durability and failure mechanisms. Such evaluations should be conducted under conditions that closely mimic real-world operation, including variations in pH, salinity, and natural organic matter content, as simplified laboratory tests may fail to capture critical degradation pathways [99].

Long-term performance loss can arise from several overlapping decay mechanisms. Reversible decline is usually associated with surface fouling, pore blockage, cake-layer formation, or temporary adsorption of organic matter and inorganic salts, which mainly reduce flux or permeance and may be partly recovered by cleaning. Irreversible decline reflects more permanent changes in the membrane, including oxidation of the polymer backbone, loss or passivation of reactive groups, radical-site depletion, swelling-induced pore changes, active-layer compaction, delamination from the support, or leaching of redox/catalytic components. These mechanisms may affect hydraulic and chemical performance differently; for example, a membrane may recover most of its flux after cleaning but still lose oxidative activity because active sites have been consumed or chemically modified. Therefore, long-term performance should be evaluated by tracking flux, permeance, pressure increase, pollutant conversion, ROS generation, oxidant utilization, and membrane chemistry before and after operation. This distinction between reversible fouling and irreversible aging is essential for identifying the dominant decay pathway and for designing suitable regeneration strategies [100].

Long-term operation under realistic wastewater conditions requires stability evaluation beyond short-term removal tests. Reactive polymeric membranes should be assessed over repeated fouling–cleaning–regeneration cycles using feeds containing natural organic matter, salts, suspended solids, and representative micropollutants. In such tests, performance recovery should be reported using both hydraulic indicators, such as flux or permeance recovery, and chemical indicators, such as retention of ROS generation, oxidant utilization, and pollutant conversion [100]. Regeneration strategies may include physical backwashing, relaxation, pH-adjusted cleaning, mild oxidant or reductant treatment, electrochemical reactivation, or solvent-free rinsing, depending on the membrane chemistry [92]. However, cleaning protocols must be selected carefully because aggressive oxidants, extreme pH, or repeated electrical polarization can accelerate polymer chain scission, loss of functional groups, delamination of active layers, or leaching of redox/catalytic components. Therefore, realistic stability assessment should compare membrane performance before and after multiple regeneration cycles and include membrane autopsy by spectroscopic, microscopic, and leachate analyses. This approach helps distinguish reversible fouling from irreversible polymer aging and provides a more reliable basis for predicting service lifetime in wastewater applications.

### 6.3. Byproducts and Toxicity-Aware Assessment

While reactive oxidation processes are effective for degrading recalcitrant contaminants, they can also generate transformation products that may be toxic or persistent. Therefore, evaluation of treated water must extend beyond simple removal efficiency and include a comprehensive analysis of byproducts and their potential impacts [101]. Identification of transformation products should be performed using advanced analytical techniques such as high-resolution mass spectrometry, gas chromatography mass spectrometry, or liquid chromatography–mass spectrometry. These methods enable both targeted and non-targeted screening, allowing detection of known and unknown compounds formed during treatment. Establishing a mass balance that accounts for the parent compound, identified transformation products, and mineralization endpoints is essential to ensure that no significant intermediates are overlooked [102]. For example, in bromide-containing waters, ozonation and other oxidative processes can convert bromide into bromate, a regulated and potentially harmful inorganic byproduct [103]. This example shows why byproduct monitoring must include not only organic transformation products but also inorganic oxidation products formed from background matrix constituents.

In addition to chemical analysis, toxicity assessment provides critical insight into the safety of treated effluent. Bioassays, including microbial luminescence inhibition, aquatic organism immobilization, and algal growth inhibition tests, can reveal adverse biological effects that may not be evident from chemical identification alone [104]. More specialized assays, such as those targeting endocrine disruption or mutagenicity, further enhance understanding of potential health risks. Importantly, the toxicity of treated water should always be compared with that of the untreated influent to evaluate whether treatment results in a net reduction or increase in hazard [105].

A practical toxicity-aware monitoring strategy should therefore combine chemical screening with biological endpoints at multiple treatment stages. At a minimum, influent, treated effluent, membrane-only controls, oxidant-only controls, and regenerated-membrane effluents should be compared to distinguish true reactive-membrane effects from background oxidation or matrix reactions. Targeted analysis can be used for known transformation products, while suspect and non-target screening by high-resolution mass spectrometry can reveal unknown intermediates. These chemical data should be interpreted together with toxicity assays, because the complete disappearance of the parent contaminant does not necessarily indicate detoxification. In addition, mineralization indicators such as total organic carbon, chemical oxygen demand, or inorganic ion release can help determine whether oxidation proceeds beyond partial structural transformation. This combined approach provides a more reliable assessment of treatment safety than parent-compound removal alone.

The nature of byproducts is strongly influenced by the type of oxidant employed. Different reactive species lead to distinct transformation pathways, and thus different toxicity profiles. For instance, selective oxidants may generate fewer harmful byproducts compared to highly non-selective radicals, although this is system-dependent. Particular attention must be paid to systems involving halogen species or ozone, where well-known disinfection byproducts such as trihalomethanes, haloacetic acids, or bromate may form [103]. Aligning performance evaluation with regulatory frameworks is therefore essential. Many regulatory bodies now impose limits not only on individual contaminants but also on classes of transformation products, requiring comprehensive reporting of total concentrations [106].

Ultimately, the implementation of reactive membrane technologies must adhere to a “no net harm” principle, whereby the reduction in target pollutants does not come at the expense of increased toxicity or formation of persistent byproducts [107]. Achieving this goal requires an integrated assessment combining chemical analysis, toxicity testing, and regulatory compliance. Adoption of standardized protocols for transformation product identification and toxicity evaluation will be crucial for enabling reliable comparison across studies and for advancing the safe deployment of reactive membrane systems in real-world water treatment applications [108].

## 7. Standardization: Metrics, Reporting, and Benchmarks

Uniform metrics and standardized reporting are essential for the rigorous evaluation and comparison of oxidative filtration systems. In the absence of clearly defined benchmarks, reported performance often reflects idealized laboratory conditions that do not translate to real-world applications, a phenomenon frequently described as deionized water optimism. To address this limitation, a structured reporting framework and the use of representative challenge conditions are necessary to ensure transparency, reproducibility, and comparability across studies [87].

### 7.1. Minimum Reporting Checklist for Oxidative Filtration Papers

**Figure 9:**
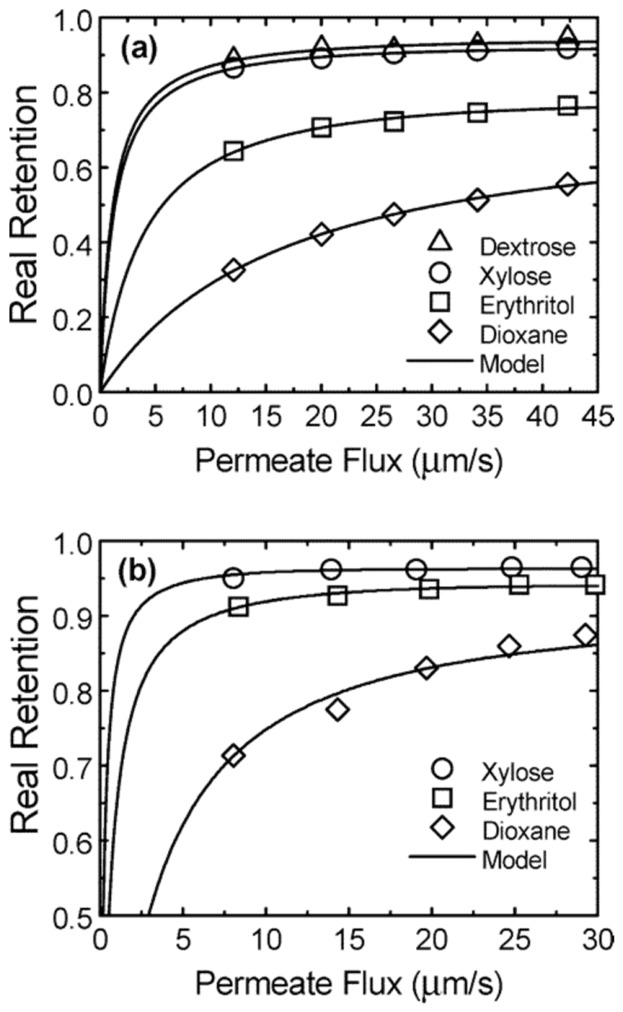
Real retention of the organic solute tracers as a function of permeate water flux for (**a**) NF-270 membrane and (**b**) NF-90 membrane. The symbols represent experimental data for the indicated organic solute tracers, while the solid lines represent the pore transport model predictions. Feed solution contained 20 mg/L of organic tracer (as TOC) in deionized water. Other experimental conditions were as follows:  cross-flow velocity = 30.4 cm/s, pH ≈ 6.0, and temperature = 20.0 °C. The permeate flux was varied by changing the applied pressure. Reproduced from Ref. [110] with permission from ACS.

A comprehensive reporting protocol should include detailed information on membrane characteristics, operational parameters, and performance metrics. Membrane properties must be clearly defined, including material composition, configuration, pore size or molecular weight cutoff, and the presence of any active or catalytic layers. Permeation performance should be reported as flux or permeance under specified pressures, with measurements provided for both clean water and reactive operating conditions [109]. Reporting flux without the corresponding driving pressure limits comparability; therefore, normalization against pressure is essential. In parallel, the characteristics of target contaminants must be explicitly stated, including initial concentrations, water matrix composition, and spiking conditions. Removal performance should be expressed not only as percentage removal but also in terms of absolute concentrations before and after treatment, distinguishing between physical rejection and chemical degradation, as transport-based retention alone does not reflect reactive removal mechanisms (Figure 9) [110].

Equally important is the quantification of oxidant usage and efficiency. Studies must specify the type and dosage of oxidants employed, whether chemical, photochemical, or electrochemical, and report these values in standardized units relative to treated volume. In addition, the extent of oxidant consumption should be determined to evaluate utilization efficiency, as unreacted oxidant represents an energetic and economic inefficiency [111]. For electrochemical systems, parameters such as current density, applied potential, and charge per unit volume should be reported alongside energy-based metrics such as EEO. These standardized metrics provide a common basis for comparing systems with different operational modes. Furthermore, identification of the dominant reactive oxygen species responsible for pollutant degradation is critical for mechanistic understanding. This requires the inclusion of diagnostic evidence, such as scavenger experiments, spectroscopic measurements, or probe-based assays, to substantiate the proposed reaction pathways [112].

Comprehensive reporting must also extend to byproduct analysis and process sustainability. Transformation products formed during oxidation should be identified and, where possible, quantified using advanced analytical techniques. Even in cases where no significant byproducts are detected, the analytical methods and detection limits should be explicitly stated to ensure transparency. Operational parameters such as total treatment duration, processed volume, flow conditions, and cleaning or regeneration cycles must also be included [113]. The evolution of membrane performance over time, particularly flux recovery and retention of removal efficiency, provides essential insight into long-term stability. Equally critical is the detailed characterization of the feed water matrix, including pH, ionic strength, dissolved organic carbon, turbidity, and the presence of co-contaminants. Without such information, performance metrics cannot be meaningfully interpreted or compared across studies, as matrix effects can significantly influence oxidation pathways and overall efficiency [114].

The adoption of a standardized reporting template ensures that all critical parameters are consistently documented, thereby facilitating cross-study comparisons and reducing ambiguity. Incomplete reporting, such as omission of oxidant dose, flux conditions, or water composition, can lead to misleading conclusions and hinder the development of a coherent knowledge base. By systematically pairing performance metrics with operating conditions and material properties, researchers can establish reproducible benchmarks that accurately reflect system capabilities [115]. To facilitate consistent and transparent reporting across studies, a standardized checklist of key parameters, units, and reporting practices is summarized in Table 2, providing a practical template for oxidative filtration research.

### 7.2. Standardized Challenge Matrices for Realistic Performance Evaluation

In addition to standardized reporting, the use of representative test conditions is essential for evaluating membrane performance under realistic scenarios. Synthetic “challenge matrices” that mimic the chemical complexity of natural and engineered water systems provide a practical approach for bridging the gap between laboratory studies and field applications. These matrices are designed to incorporate key components that influence oxidative processes, including natural organic matter, inorganic ions, and target micropollutants [116].

**Figure 10:**
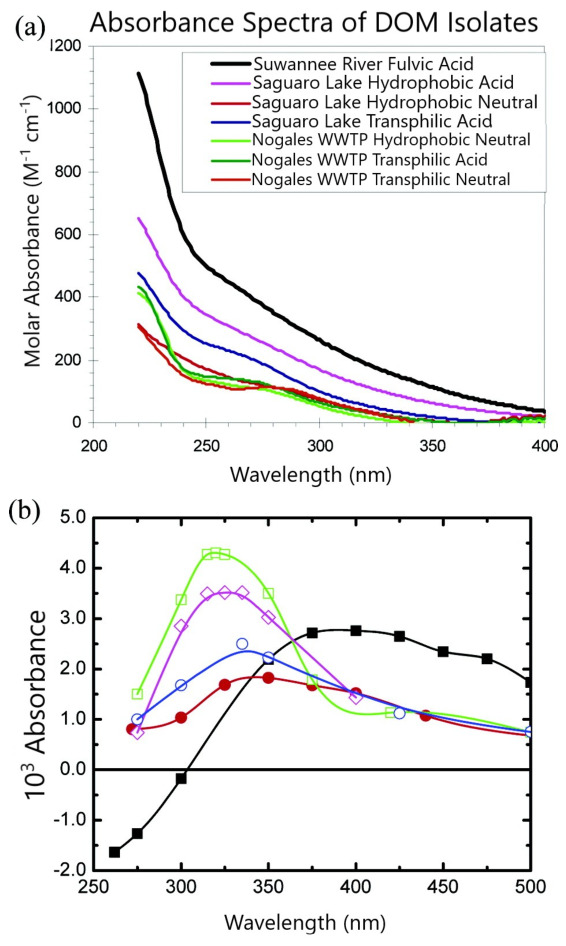
(**a**) Molar absorbance spectra for DOM isolates. (**b**) Transient absorption spectra for hydroxyl radical reaction with DOM isolates in 0.10 M phosphate-buffered, N_2_O-saturated water at pH 7.0. All spectra taken ∼50 μs after electron pulse and normalized to a dose of 5.0 Gy. (**▪**) Suwannee River Fulvic Acid, (red filled circle) Saguaro Lake Hydrophilic Neutral, (green open square) WWTP Hydrophilic Neutral, (blue open circle) WWTP Transphilic Neutral, and (fuchsia diamond) WWTP Transphilic Acid isolates. Reproduced from Ref. [127] with permission from ACS.

Natural organic matter is a critical component of such matrices, as it is ubiquitous in surface waters and treated effluents and plays a significant role in modulating oxidation reactions through light absorption and radical scavenging processes, as illustrated by spectroscopic characterization of DOM isolates (Figure 10) [127]. Including representative concentrations of humic and fulvic substances allows assessment of competitive reactions, radical scavenging, and fouling behavior. Similarly, the inclusion of dissolved ions such as calcium, magnesium, and chloride reflects the ionic strength and hardness of real waters, which can significantly influence radical chemistry and membrane performance. In systems involving ozone or advanced oxidation processes, the presence of bromide is particularly important, as it can lead to the formation of regulated byproducts such as bromate under ozonation conditions [123] (Figure 10).

Beyond their individual effects, these matrix constituents can also shift the dominant ROS pathway during oxidative filtration. Natural organic matter can consume oxidants and quench HO^•^ and SO_4_^•−^, while bicarbonate and carbonate can convert highly reactive HO^•^ into carbonate radicals, which are generally less reactive but more selective toward electron-rich compounds. Chloride may form chlorine-derived radicals, altering degradation pathways and increasing the possibility of halogenated transformation products. Competing ions can also modify membrane swelling, surface charge, pollutant partitioning, and oxidant transport near the reactive layer. Therefore, ROS selectivity observed in deionized water may not represent behavior in real wastewater, and matrix composition should be treated as a mechanistic variable rather than only as background information.

Control of pH and alkalinity is another essential factor, as these parameters directly affect reaction kinetics and radical stability. The incorporation of bicarbonate to simulate natural buffering capacity is especially relevant in systems where carbonate species can act as radical scavengers. Additionally, the presence of suspended solids or turbidity can be included to evaluate the combined effects of physical fouling and catalytic activity under realistic conditions. These components collectively create a testing environment that challenges both the separation and reactive functionalities of the membrane [128]. The selection of target micropollutants within the challenge matrix should reflect a range of physicochemical properties, including molecular size, charge, and hydrophobicity. A representative mixture may include neutral, anionic, and hydrophobic compounds to capture diverse interaction mechanisms with both the membrane and reactive species. The use of environmentally relevant concentrations ensures that observed performance is applicable to real treatment scenarios. Where relevant, additional co-contaminants such as nutrients or model dyes can be incorporated to evaluate system robustness and multifunctionality [122].

By adopting standardized challenge matrices, researchers can generate comparable datasets that reflect realistic operating conditions. This approach aligns with established practices in related fields, where synthetic feeds are routinely used to benchmark performance across technologies. Detailed reporting of matrix composition, including all constituents and preparation methods, enables reproducibility and facilitates the development of shared performance databases. Over time, such standardized testing frameworks will allow the construction of performance envelopes that define system capabilities across a range of conditions [126].

In summary, the integration of standardized reporting protocols with representative challenge matrices is essential for advancing the field of oxidative membrane technologies. These practices address current limitations associated with inconsistent metrics and idealized testing conditions, thereby enabling more reliable comparisons and accelerating the translation of laboratory innovations into practical water treatment solutions.

## 8. Scale-Up and Deployment: Pilot/TRL and Process Integration

Translating intrinsic reactive polymer membranes from laboratory concepts to real-world applications requires a shift from material-centric evaluation to system-level assessment. At this stage, performance is governed not only by intrinsic reactivity but also by hydrodynamics, mass transfer, stability under complex water matrices, and integration within existing treatment trains. This section, therefore, examines how oxidative filtration technologies perform under pilot conditions, defines practical technology readiness pathways, and discusses the engineering, economic, and regulatory factors that ultimately determine their feasibility for full-scale deployment.

### 8.1. Pilot-Scale Evidence for Micropollutant Treatment Trains

#### 8.1.1. From Lab Performance to Pilot Reality

Laboratory investigations of micropollutant elimination sometimes depict unrealistic scenarios that do not accurately reflect full-scale treatment processes. In bench systems, contact durations are clearly established, matrices are optimized, and oxidant demand is regulated; conversely, actual waters present brief and varied residence times, competing natural organic matter (NOM), inorganic ions, variable hydraulics, and persistent fouling, all of which impact the estimated reaction rate and pollutant elimination [129,130]. This difference is a persistent pattern in the literature on emerging-contaminant treatment, in which full-scale and pilot systems exhibit behavior that differs from that of laboratory reactors because transport and reaction occur simultaneously under actual matrix constraints. A significant perspective on this discrepancy is that laboratory tests generally focus on chemistry in isolation, while pilot systems incorporate chemistry with hydrodynamics and mass transfer. Despite a material exhibiting elevated radical activity or intrinsic oxidation rates, effective removal may be diminished by oxidant scavenging, inadequate mixing, boundary-layer constraints, and rapid depletion of reactive species before they interact with the target pollutant. Ullberg et al. observed that pilot-scale studies of ozonation and hybrid oxidation processes prioritize matrix effects and dynamic operation over intrinsic kinetics alone [131]. Comprehensive surveys and pilot studies indicate that new pollutants, including pharmaceuticals and pesticides, are frequently inadequately removed by conventional treatment processes, underscoring the need to assess performance under real-world conditions rather than hypothetical scenarios [132]. Analyses of micropollutant behavior underscore that efficient removal necessitates not only substantial reactivity but also resilience against fouling, competition from oxidants, and sustained operation, factors often neglected in small-scale laboratory evaluations, as concluded by Silva et al. [133].

#### 8.1.2. Established Advanced Treatment Trains as Benchmarks

**Figure 11:**
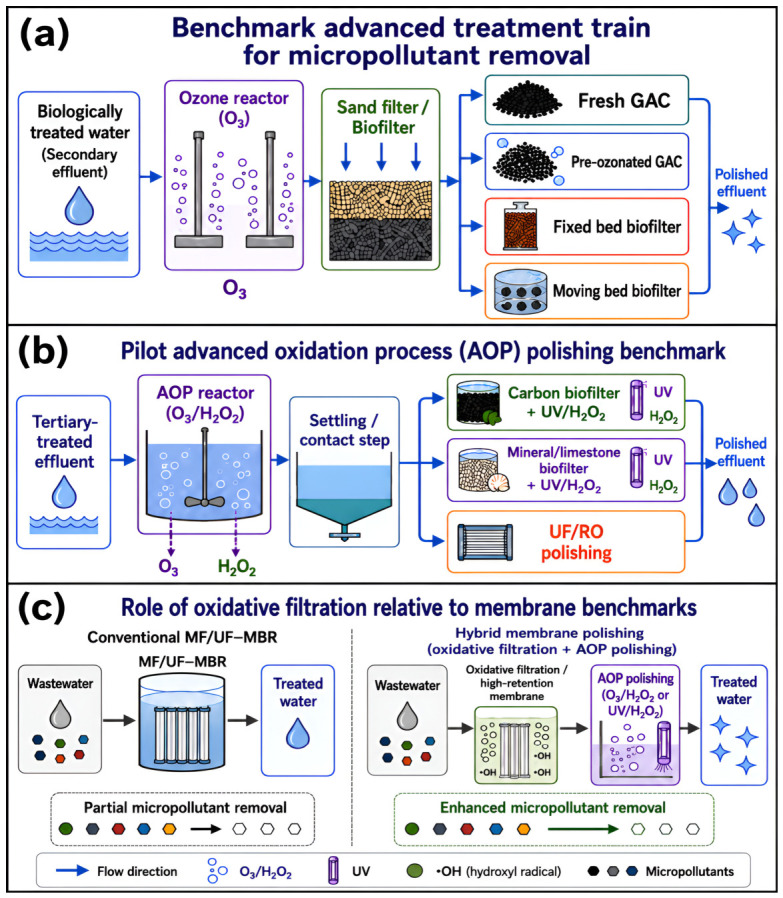
Schematic overview of established advanced treatment trains used as benchmarks for oxidative filtration. (**a**) Ozonation followed by sand filtration/biofiltration and granular activated carbon (GAC)-based polishing for micropollutant removal. (**b**) Pilot-scale AOP polishing train combining O_3_/H_2_O_2_, settling/contact treatment, biofiltration, UV/H_2_O_2_, and UF/RO polishing. (**c**) Comparison between conventional MF/UF–MBR treatment and hybrid membrane polishing, illustrating how oxidative filtration or high-retention membranes can enhance micropollutant removal when combined with AOP-based post-treatment.

Current advanced treatment systems serve as the most vital standard for evaluating oxidative filtration [131]. Ozonation, followed by biofiltration, is a recognized method for refining drinking water and tertiary wastewater, demonstrating significant efficacy against numerous trace organics due to ozone’s rapid oxidation capabilities and the biofilter’s ability to eliminate biodegradable transformation products and residual oxidant demand [134,135]. In Switzerland, Bourgin et al. reported that full-scale ozonation, as shown in Figure 11a, at a specific dosage of 0.55 g O_3_/g dissolved organic carbon (DOC), can achieve an average reduction of less than 80% for indicator micropollutants [136]. Concurrent monitoring of 550 substances corroborated a comprehensive removal exceeding 79%, yet underscored the necessity for biological post-treatment and granular activated carbon (GAC) to mitigate transformation products and ecotoxicological risks. The combination of UV/H_2_O_2_ with activated carbon is widely used as an effective polishing step, especially when broad-spectrum oxidation needs to be coupled with the adsorption of residual organics and byproducts. In these benchmark trends, the removal efficiencies for various micropollutants typically range from approximately 70% to 95%, depending on compound class, oxidant dosage, matrix quality, and post-treatment polishing. Piras et al. observed that a two-stage AOP train (O_3_/H_2_O_2_ + biofiltration + UV/H_2_O_2_) achieved 99% total micropollutant abatement in municipal wastewater and demonstrated that multi-unit configurations combining oxidation with biofiltration and activated carbon are particularly effective for potable-reuse targets without significant ecotoxicological responses (Figure 11b) [137]. Assessment is rarely reliant on a single chemical; rather, investigations generally monitor the elimination of target compounds, the reduction in DOC, and alterations in toxicity or in the formation of intermediate products.

For intrinsic reactive polymeric membranes, the design opportunity is to integrate two traditionally separate functionalities: filtration and oxidation. If successful, oxidative filtration could offer a more compact alternative to multi-unit trains, especially when space, operational simplicity, or modularity are important [138]. The conceptual advantage is not merely higher integration, but potentially better coupling between contaminant concentration at the membrane interface and local formation of reactive species. In this context, as illustrated in Figure 11c, Khan et al. emphasized that conventional ultrafiltration- or microfiltration-based membrane bioreactors (MBRs) achieve only partial micropollutant removal [139]. Hybrid systems combining high-retention membranes or AOP-based post-treatment are therefore required for near-complete elimination, which highlights the relevance of oxidative filtration as a targeted, oxidation-intensified polishing layer rather than a generic separation barrier.

#### 8.1.3. Coupling with NF/RO Systems and Concentrate (Brine) Treatment

Nanofiltration and reverse osmosis (NF/RO) serve as essential benchmarks, as they already dominate high-rejection polishing and PFAS control in many advanced treatment trains. These systems are employed either as pre-treatment to prevent fouling and preserve downstream RO performance, or as post-treatment to polish permeate for reuse applications. The literature on PFAS treatment reports that NF/RO often achieves extremely high rejection [140]. However, they also generate concentrated brine streams that must be managed carefully, and brine handling is repeatedly cited as a significant impediment to the broader deployment of membrane-based contaminant removal, as reported by Li et al. [141]. Reactive polymer membranes can integrate into these NF/RO-centered trains in numerous ways. As pre-treatment, they may minimize organic fouling and oxidize troublesome trace impurities before the pressure-driven stage, hence enhancing RO lifetime and energy efficiency. As post-treatment, they can target residual micropollutants or PFAS-related species in permeate or interstage streams, where the contaminant load is smaller but regulatory compliance is stricter [142]. In both roles, oxidative membranes are rated not by isolated removal but by how effectively they fit into the whole train and by whether they reduce the strain on downstream units [143].

At the same time, concentrated brine streams constitute a particularly promising niche for oxidative filtration, because pollutants are enriched to levels where reaction kinetics become more favorable [144]. This is especially relevant for PFAS and other persistent trace organics, since physical rejection moves the problem into a smaller-volume concentration that is difficult to address with standard AOPs. Oxidative membranes may be advantageous in this context because local enrichment at the membrane interface can overcome the low-concentration constraint that commonly limits uniform bulk oxidation, making the concentrate a potential target for destructive treatment rather than merely a confined waste stream. In this way, coupling NF/RO with reactive polymer membranes offers a dual benefit: less foulant and oxidant-sensitive feedwater for the high-pressure unit, and an integrated destruction pathway for the concentrated stream, which would otherwise pose a major environmental and operational challenge [145,146].

Beyond NF/RO coupling, reactive polymer membranes can also be integrated with biological, adsorption-based, and electrochemical treatment units. After biological treatment, they may serve as compact polishing barriers for micropollutants that are poorly biodegraded, while the upstream biological stage lowers bulk organic load and reduces oxidant demand. In adsorption–oxidation hybrids, sorptive polymer domains or added adsorbent phases can first concentrate trace contaminants near reactive sites, followed by in situ oxidation that regenerates adsorption capacity and reduces secondary waste. Electrochemical integration is particularly relevant for conductive or redox-active polymer membranes, where applied potential can regulate oxidant activation, ROS generation, and membrane regeneration during flow-through operation. These hybrid configurations may improve treatment efficiency and extend membrane lifetime, but they require careful control of energy use, byproduct formation, fouling, and compatibility with existing plant operation.

#### 8.1.4. Utility-Relevant Performance Metrics

Utilities evaluate treatment systems differently from research laboratories, and a key change from academic to utility-driven evaluation is necessary for successful scale-up. While laboratory studies frequently focus on emphasizing maximum degradation efficiency or apparent rate constants, utilities prioritize operational durability, predictability, and cost control over short-term peak performance. Intrinsic reactive polymer membranes must therefore be rated not by their best-case removal under ideal water, but by how consistently they function in real-world trains with changing influent quality, fouling, and oxidant demand [93,147].

Key performance factors for utility-relevant assessment include robustness, operational simplicity, energy demand, and maintenance frequency. Despite variations in influent composition, temperature, and organic load, robustness is demonstrated by stable removal performance over prolonged operation (weeks to months); full-scale and pilot studies of ozonation, biofiltration, and advanced oxidation-activated carbon trains demonstrate that plant adoption is ultimately determined by robustness to matrix variability rather than maximum single-point efficiency. Because systems that can be integrated into existing control platforms and operated with minimal operator intervention reduce human error and long-term operational costs, operational simplicity includes minimal reliance on precise chemical dosing, complex control logic, or highly specialized maintenance [131].

Energy demand should be expressed in readily recognizable units, such as kWh.m^−3^ or E_EO_, to permit direct comparison with UV-based and other AOP standards [148]. High micropollutant removal is less desirable if it comes at the cost of prohibitive energy input or oxidant consumption adjusted per unit of treated water and removal achieved. Maintenance frequency, encompassing cleaning intervals, fouling rates, and the simplicity of physical cleaning versus chemical/oxidative regeneration, is equally significant, as frequent or extensive cleaning might offset gains from high removal if it increases downtime, chemical use, or membrane damage [149]. Importantly, utilities frequently choose consistent, moderate performance over unstable, high efficiency. A system that reliably achieves 85% removal over long periods is often more valuable than one that sometimes reaches 99% removal but suffers from performance drift, fouling, or complex control requirements [150]. Intrinsic reactive polymer membranes will consequently need to exhibit stable, predictable behavior under realistic water matrices and operational conditions if they are to advance beyond proof-of-concept demonstrations and into utility-facing deployment.

Although the literature shows that reported values vary significantly with reactor design, oxidant dose, and water matrix, E_EO_ values provide a useful tool for assessing the energy intensity of advanced oxidation systems. E_EO_ values as low as 0.058–0.105 kWh m^−3^ order have been reported for full-scale UV-AOP flow-through reactors, showing comparatively efficient performance under optimal conditions [151]. Broader AOP comparisons, on the other hand, demonstrate that E_EO_ values can increase significantly under less favorable operating conditions; published values include 1.68 for O_3_/MnO_2_ under 365 nm and 2.01 for UV/H_2_O_2_/Fe^3+^ under 254 nm [68]. Meanwhile, full-scale municipal MBRs report membrane-related energy usage of 0.5–0.7 kWh m^−3^ [152]. These figures demonstrate how sensitive E_EO_ is to process configuration, and if it is to be considered a reasonable benchmark for reactive membrane systems, it should be provided alongside matrix and dose conditions.

Because no single metric is sufficient to judge utility relevance, process comparisons should combine removal efficiency with hydraulic productivity, energy demand, oxidant or electrical input, and long-term stability. A system that achieves high micropollutant removal only at low flux, high oxidant dose, or short operating time may be less practical than a system with moderate but stable removal under realistic flow and matrix conditions. Therefore, quantitative benchmarks are needed to compare established membrane processes, conventional AOP trains, electrochemical systems, and emerging reactive polymer membranes on a common basis. Table 3 summarizes representative performance ranges and practical limitations for these treatment options.

#### 8.1.5. Acceptable Performance and Limitations

“Acceptable performance” for oxidative filtration should be determined based on actual deployment requirements rather than optimal laboratory metrics [131]. An achievable objective for a micropollutant-focused oxidative membrane would entail consistent removal rates of 80–90% for priority compounds across representative water matrices, alongside stable flow, minimal pressure drop, and controlled generation of transformation products. Performance must be replicable during multiple cycles and prolonged operation, rather than solely during a brief test, as utilities prioritize consistent, predictable behavior above temporary efficiency surges. Huang et al. [162] illustrated that nanofiltration membrane assessment can be significantly compromised by inconsistent testing protocols and external variables, such as dead volume, rotor effects, and adsorption, highlighting the necessity of standardized, full-flow, long-duration testing to ensure equitable comparisons with performance benchmarks.

Real matrices are crucial, as NOM, carbonates, chlorides, bromides, and co-contaminants can significantly influence oxidant demand and radical pathways. A membrane that excels in deionized water may exhibit markedly different behavior in secondary effluent, surface water, or saline concentrations, where fouling, oxidant competition, and intricate hydraulics significantly influence performance [139]. Consequently, satisfactory performance must encompass not only the elimination of parent compounds but also demonstrable stability against fouling, chemical degradation, and realistic hydrodynamic conditions. Cran et al. [163] demonstrated that rigorous chemical cleaning and oxidation in polymer-based systems can result in significant alterations to membrane structure and swift deterioration. At the same time, Scholes [164] underscored that industrial-scale membrane pilot plants for gas separation consistently fail when flow-field design, pressure equilibrium, and mechanical contact are inadequately managed. Both findings highlight the necessity for oxidative filtration membranes to be validated under realistic operational stresses to be deemed robust.

In the context of oxidative filtering, the notion of acceptability must also encompass a safety aspect. It is not enough to eliminate the parent contamination if the procedure generates hazardous byproducts or changes membrane integrity. Pilot failures in advanced oxidation and hybrid membrane systems frequently stem from a limited set of persistent issues: firstly, activity diminishes due to fouling or chemical degradation, particularly when active sites are subjected to oxidants, natural organic matter, or reactive radicals for extended durations; secondly, transformation products may significantly diverge from those identified in simplified laboratory experiments, with some potentially exhibiting greater persistence or toxicity than the original compound; and thirdly, scaling up can uncover hydraulic challenges that remain undetected in smaller cells, such as pressure drop accumulation, channeling, uneven flow distribution, and localized dead zones. In this light, Scholes [164] and Weiland et al. [165] explained that the design and flow-field configuration of membrane systems strongly influence fluid distribution, pressure balancing, and mechanical contact, so that inadequate module-level engineering can impair otherwise good material performance.

The aforementioned failures emphasize a deeper point: treatment performance is not determined solely by material activity but also by how the active layer is incorporated into a module and operated in a real train. A membrane that demonstrates robust chemistry may still underperform if mass transport is poor, if the hydraulic design generates maldistribution, or if the active chemistry is lost too soon under real-water conditions. Likewise, short-term studies generally overlook long-run cleaning, regeneration, and failure analysis, leaving the most essential deployment concerns unaddressed. In the case of oxidative-driven membrane processes (ODMPs), Cath et al. [166] pointed out that operating conditions and membrane properties play a more critical role in mass transport and process performance than in conventional pressure-driven systems, and that the lack of standardized test protocols, such as inconsistent temperatures, draw-solution compositions, flow rates, and applied pressures, leads to data-reliability problems and misleading comparisons. This replicates the difficulties reported by Huang et al. [162] for nanofiltration, where the absence of standardized separation-rate indicators and inadequate control of external factors, such as dead volume and adsorption, have made published performance data confusing and hard to assess.

The gap, therefore, is not merely better chemistry; it is integrated, system-level validation. Pilot-relevant oxidative filtering systems must show that they can sustain function in actual matrices, retain activity after cleaning and regeneration, keep hydraulic losses and flow maldistribution tolerable, and avoid undesirable byproducts or structural deterioration. Until those criteria, performance, robustness, safety, and standardized test rigor, are met, lab-scale success should be taken as proof of concept rather than evidence of readiness for deployment.

### 8.2. TRL Framework for Reactive Polymer Membranes

#### 8.2.1. Current Maturity of the Field

Intrinsic reactive polymer membranes for oxidative filtration are currently at an early stage of development [167,168]. In TRL terminology, most concepts remain around TRL 2–3, where the primary achievements are proof of concept, material synthesis, and small-scale laboratory validation. A limited number of investigations have advanced to TRL 4–5, often by testing in realistic water matrices. However, sustained pilot validation remains rare for membrane systems that combine filtration with in situ oxidation. This is similar to the broader membrane water-treatment literature, where revolutionary membrane concepts frequently move slowly because the problem is not only performance, but also stability, fouling tolerance, and manufacturability, as analyzed by Tian et al. [169].

The main reason for the restricted maturity is that oxidative filtration is not merely a materials issue, as it demands the simultaneous management of oxidant compatibility, active-site persistence, byproduct generation, and hydraulic stability under prolonged exposure to actual water. Reviews on membrane oxidation and pre-oxidation consistently emphasize that long-term exposure can damage membranes, alter rejection, and raise compatibility concerns that only become apparent during prolonged operation [100,169].

#### 8.2.2. Defining TRL Gates for Oxidative Filtration Systems

A useful TRL framework should specify explicit gateways between laboratory concept, real-matrix validation, pilot readiness, and demonstration [169]. At TRL 3 to 4, the membrane should exhibit combined filtration and oxidation in a controlled configuration, with short-term evidence for the target reactive oxygen species pathway and basic proof that oxidation occurs with permeation rather than solely in the bulk feed [170]. At this stage, the important requirement is mechanistic credibility, not long-duration endurance.

At TRL 4 to 5, the system should be tested in real matrices such as secondary effluent, surface water, or saline concentrate, where natural organic matter, ions, and competing solutes challenge both transport and reactivity. This is often where systems fail, because pre-oxidation and membrane treatment experiments reveal that oxidant compatibility, fouling behavior, and byproduct generation can alter substantially in realistic water [100]. At TRL 5 to 6, the membrane and module should be manufacturable over a larger area, with pressure drop, flow distribution, and module-scale operation characterized under representative conditions [171]; by TRL 6 to 7, long-term operation, cleaning protocols, and regulatory-relevant byproduct performance should be demonstrated.

Numerous advanced materials, including covalent organic frameworks (COFs), metal–organic frameworks (MOFs), and conductive polymers, have demonstrated remarkable potential in laboratory research; however, their commercial-scale application is constrained by common obstacles. In the case of MOFs, a persistent challenge is framework flexibility, which can obscure the effective pore size and compromise their application as precise molecular-sieve membranes. In contrast to rigid zeolites, metal–organic frameworks (MOFs) lack a fixed pore size; for example, ZIF-8 has a crystallographic aperture of 3.4 Å but an effective pore size of approximately 4.3 Å [172]. This discrepancy has prompted the development of strategies such as mixed-linker synthesis, rapid thermal treatment, and electrochemical deposition to limit flexibility and enhance separation selectivity [173]. Simultaneously, significant progress is still required to establish efficient, ubiquitous, and economical methods for producing ultrathin MOF/COF membranes [174]. While contrast diffusion growth, layer-by-layer assembly, and metal pre-functionalized substrates can mitigate heterogeneous nucleation and customize growth, these techniques often exhibit limited applicability across various metal–organic frameworks (MOFs) and substrates and face challenges with uncontrolled crystal growth and intricate MOF structures [175]. The in situ growth of COFs is particularly difficult due to slow reaction kinetics and elevated temperature requirements, which hinder crystallization and adherence, particularly on polymer substrates [176]. Interface-assisted strategies that produce ultrathin, even few-layer, 2D MOF/COF films are promising; however, their practical application is limited by weak film-support adhesion during pressure-driven operation. Recent research has concentrated on superspreading surfaces and mussel-inspired surface modifications to enhance adhesion and prevent transfer-induced defects. The comparatively high cost of MOF/COF synthesis and processing further hampers large-scale implementation.

Conductive polymers encounter similar challenges regarding scalability and processability. Although electrically conductive membranes provide enhanced permeate flux, antifouling properties, and superior separation of charged species, their efficacy remains constrained by fouling and by the challenges of optimizing the hydrophilic/hydrophobic balance and porous architecture to attain desired flux and rejection rates [177]. Current approaches to modify morphology and pore architecture are frequently expensive, time-consuming, or insufficiently reproducible at pilot or plant scale. The principal obstacles to the extensive commercial use of COFs, MOFs, and conductive polymers in oxidative filtration and associated reactive membrane systems are synthetic, structural, and processing constraints, rather than a deficiency in intrinsic activity [178].

#### 8.2.3. Stability Under Realistic Conditions

Stability is the primary obstacle for reactive polymer membranes [168]. Real water contains NOM, bicarbonate, chloride, bromide, and other compounds that can block the desired reactive pathway, compete for oxidants, or divert the chemistry onto nonproductive routes. In addition, oxidant exposure can erode active sites, induce radical quenching, or modify polymer structure over time, so measured reactivity may fall even when flux remains acceptable. For that reason, stability should be split into three independent questions: does the membrane retain its transport properties, does it maintain its reactivity, and does it avoid unacceptable leaching or structural damage? Pre-oxidation and membrane-aging studies demonstrate that oxidative exposure can induce progressive breakdown, whereas radical-rich environments can also promote chain scission or grafting side reactions in sensitive polymers. In practical terms, a reactive membrane should only be called stable if its active chemistry endures the same matrix circumstances under which it is meant to work [179].

Current research also argues that stability must be examined from a practical rather than a purely chemical perspective. Shi et al. [14] suggested that multi-cycle studies and long-time filtration best assess catalytic membrane stability, because deactivation commonly occurs due to pollutant adsorption, oxidation intermediates, chemical changes in the membrane, or leaching of active species. Wang et al. [180] further showed that grafting radical scavengers can greatly increase membrane chemical stability, even under severe oxidative testing, demonstrating that molecular design can be employed to resist oxidative damage rather than simply compensating for it after failure.

#### 8.2.4. Cleaning and Regeneration Strategies

The cleaning approach should be incorporated into the membrane design rather than treated as an afterthought [181]. Physical cleaning eliminates accumulated foulants, whereas functional regeneration restores the membrane’s reactive state, which may require reactivation of radicals, redox centers, or catalytic moieties. In oxidative systems, the harshest cleaning procedure is not necessarily the best, because aggressive chemical cleaning may recover flux while simultaneously degrading the polymer or deactivating reactive sites. The literature on fouling control suggests that cleaning must be tailored to the membrane chemistry and the nature of the foulants. Yalcinkaya et al. [182], for example, underlined that membrane technology is particularly effective for oily wastewater because it combines high separation performance with operational simplicity. However, they also stress that fouling and self-cleaning surface design are crucial to long-term performance. This confirms the idea that durable operation depends on regulating foulant accumulation within the membrane architecture, rather than treating cleaning as a separate troubleshooting process.

For intrinsic reactive membranes, regeneration should therefore be benchmarked by two outcomes: recovery of hydraulic performance and recovery of oxidative function. A membrane that recovers flow but loses activity is not genuinely regenerated in the context of oxidative filtration. Kim et al. [181] provided a useful example from dynamic membranes: NaOCl clean-in-place restored most of the original flux and improved removal performance compared with NaOH, while periodic re-deposition of the dynamic layer extended service life. This shows that the best cleaning protocol is the one that restores function without eroding the active structure. Long-term research should also describe whether regeneration changes the membrane itself. Liu et al. [183] demonstrated that long-term testing can reveal only a minor performance decline when membrane chemistry and morphology are designed for stability, suggesting that regeneration success is not simply a matter of cleaning intensity but of compatibility between the membrane structure, the foulant, and the cleaning chemistry. Taken together, these results establish a practical guideline for reactive polymer membranes: regeneration must preserve both transport and reactivity, and the best technique is the least aggressive one that reliably restores both.

#### 8.2.5. Module Integration, Hydraulics, Safety, and Regulatory Compliance

Module design is an important TRL gate (TRL 3) because scale-up reveals hydraulic concerns that are not obvious at the lab scale. Pressure drop, channeling, dead zones, and disproportionate distribution can all diminish effective residence time and create local regions where oxidation is either wasted or overly intense, especially when the reactive layer must operate under crossflow or in a densely packed module design. Scaling from flat-sheet tests to hollow-fiber or spiral-wound formats provides extra complexity in packing density, flow homogeneity, and cleaning accessibility. The literature consistently reveals that modularity is one of the benefits of membrane systems, but it also means that module engineering is interdependent of material performance [184]. For oxidative filtration, the challenge is therefore not only whether the membrane works, but if the module allows the reactive layer to operate evenly and stably at scale, without excessive trans-membrane pressure, uneven flow, or inaccessible cleaning zones [100].

At the same time, safety and regulatory compliance form a parallel TRL barrier, because oxidative membrane systems can develop or concentrate regulated byproducts depending on oxidant chemistry and water composition. In particular, oxidant use can raise concerns about bromate, chlorate, residual oxidants, and transformation products, and pilot and review work on advanced oxidation and pre-oxidation consistently treats byproduct control as a key deployment requirement, not a peripheral issue [137]. Regulatory acceptability also hinges on worker safety, oxidant management, and confirmation that the system is not causing additional health or environmental concerns while eliminating toxins.

Concluding, a reactive membrane will likely not be considered beyond the pilot stage unless it can demonstrate compliance with byproduct limitations, robust operation under realistic matrices, and safe handling practices. In practice, this means that regulatory performance standards and safe, well-designed modules must be incorporated into the TRL definition itself, because a system that fails to comply or exhibits unsafe hydraulics cannot be regarded as deployment-ready, even if its removal efficiency is good.

## 9. Interdisciplinary Accelerators: AI, Digital Twins, and LCA and TEA-Informed Design

Advancing oxidative filtration beyond proof-of-concept requires the integration of data-driven design, system-level modeling, and sustainability assessment. Emerging tools such as artificial intelligence, digital twins, and life cycle analysis provide a unified framework to connect material properties with process performance, operational control, and environmental impact. By enabling predictive design, real-time optimization, and circular engineering strategies, these interdisciplinary approaches offer a pathway to accelerate the development of robust, scalable, and sustainable reactive membrane technologies.

### 9.1. AI for Membrane and Process Design

#### 9.1.1. Why AI Is Needed in This Field?

The existing design of reactive polymeric membranes for oxidative filtration predominantly relies on trial-and-error methods, with limited predictive capability for the correlation between structure, transport, reactivity, and stability. As the domain of polymer chemistries, structures, and operational circumstances expands, comprehensive experimentation becomes unfeasible; hence, AI and machine learning can assist by identifying trends within sparse, heterogeneous data and simplifying targeted design [185]. Recent evaluations of PFAS and micropollutant remediation emphasize the necessity for integrated data-model workflows to transition from ‘single-hit’ laboratory membranes to scalable, regulation-compliant solutions [146,153]. In oxidative filtration, artificial intelligence is particularly pertinent as performance relies on an interconnected array of descriptors: membrane shape, redox/radical content, cross-linking density, fouling propensity, and reaction kinetics within realistic matrices [142]. Instead of considering them as distinct tuning parameters, AI can identify concealed relationships and trade-offs, facilitating the design of membranes that concurrently optimize permeance, conversion, and longevity. This is a rising trend in advanced membrane and AOP analysis, rather than traditional empirical screening.

#### 9.1.2. ML for Polymer Material Discovery and Process Optimization

Machine-learning methods can expedite polymer discovery by predicting essential membrane characteristics, like permeance, rejection, and fouling resistance, directly from chemical descriptors [186,187]. These descriptors may encompass monomer identity, functional groups, backbone polarity, cross-linking motifs, and anticipated surface charge or hydration, which are subsequently employed to train models that correlate with macroscopic transport and compatibility in oxidative settings [188]. Recent work on nanocomposite and advanced polymeric membranes has shown that regression and graph-based models can reasonably predict permeability and selectivity even with inadequate training data [189]. For intrinsic reactive membranes, the challenge is to extend these approaches to reactivity-oriented outputs: not just water flux and solute rejection, but also radical stability, ROS production capacity, and sensitivity to oxidant scavengers. By encoding redox-active motifs, donor-acceptor interactions, and probable aging pathways into the descriptor set, ML models can search for polymer compositions expected to sustain both catalytic activity and mechanical integrity under oxidative stress. This reframes material discovery from finding a high-performance polymer to finding a polymer that satisfies transport, reactivity, and stability constraints.

Beyond membrane chemistry, AI can also optimize operating conditions, fouling management, and oxidant dosing across oxidative filtering systems. In practice, flux, pressure, cross-flow velocity, oxidant concentration, and matrix composition interact nonlinearly to determine contaminant residence time, ROS yield, and polymer aging, so merely empirical adjustment is uneconomical [169]. Machine-learning controllers and surrogate models have already been applied to ozonation, UV/H_2_O_2_, and membrane-AOP hybrids to forecast removal efficiency and modify oxidant dose in response to varying water quality. Araujo et al. [190] further showed that kinetic and isotherm adsorption models can reproduce batch behavior and predict industrial adsorption-column performance, which is useful here because it illustrates how data-driven or semi-empirical models can bridge laboratory observations and process-scale design. For oxidative filtration, ML can similarly help identify optimal setpoints for flow, oxidant input, and cleaning frequency that minimize energy and chemical expenditure while maintaining target removal, and predict fouling trajectories and transmembrane-pressure rise for proactive intervention. This is especially beneficial for incorporating oxidative membranes into existing treatment trains, where the system must remain robust to feed fluctuations while still meeting regulatory requirements [153].

#### 9.1.3. New Descriptors and Benchmark Datasets for Oxidative Filtration

Oxidative filtering requires a customized collection of AI-ready indicators that capture the coupling among reaction, transport, and degradation to go beyond basic membrane-property descriptions. Dimensionless reaction-transport measures, such as effective Damköhler numbers per membrane layer, ROS-selectivity indicators, and aging-resistance factors obtained from accelerated-oxidation studies, should be useful descriptors [191]. Furthermore, fouling-reactivity coupling descriptors, such as contaminant enrichment at the membrane surface, NOM scavenging capacity, and fouling-induced shifts in reaction-zone location, are crucial because they help differentiate between materials that perform well in ideal batch tests and those that continue to perform well under realistic distributed residence time conditions [192].

The lack of standardized, shareable data linking membrane characteristics, operating conditions, and performance measures across many systems is a primary obstacle to AI adoption in this industry, underscoring the importance of a benchmark dataset [193]. Membrane architecture, polymer chemistry, active-site concentration, feed-matrix composition, flux, rejection, conversion, oxidant use, byproduct profiles, and pressure or aging data over time should all be included in a usable dataset [194]. To report oxidant consumption, ROS diagnostics, and byproduct toxicity in a consistent manner, even when diverse reactor designs are employed, the community should also agree on metadata templates. A small collection of representative systems covering stable-radical membranes, redox-active polymer layers, and porous conjugated catalytic membranes would be a useful place to start. This way, the dataset would cover the entire reactivity-transport-stability space and eventually support more broadly applicable AI models.

Because they serve as a link between mechanical understanding and predictive design, these descriptors and datasets are crucial. ML models can be used to filter materials, determine operating windows, and convert mechanistic insights into useful design guidelines for oxidative filtration by storing process-relevant characteristics rather than relying solely on static membrane parameters.

### 9.2. Digital Twins in Water Systems

#### 9.2.1. Concept of Digital Twins and Application in Water Treatment

A digital twin is a dynamic, virtual model of a physical system that is used for control, monitoring, and simulation. It is constantly updated with real-time sensor data. By connecting process models, instrumentation, and operational logs into a unified computing environment, digital twins in water treatment enable engineers to test control techniques, investigate scenarios, and predict failures without interfering with the actual plant. This idea is particularly potent for oxidative filtration, as the system combines transport, reaction, and aging, allowing the twin to incorporate these effects into a single predictive framework and facilitate quicker membrane and module design iterations [195,196].

In reality, digital twins are interactive replicas that can be examined and adjusted as circumstances change, rather than merely being static representations. In today’s water infrastructure, where demand fluctuations, climate unpredictability, and regulatory pressure necessitate quick, adaptive responses rather than fixed-setpoint operation, this is becoming increasingly crucial. Kazakovtseva et al. [197] demonstrated this logic by combining neural networks with high-accuracy numerical simulations of electroconvection, employing dimensionless similarity criteria such as Reynolds and Péclet numbers to develop rapid surrogate models for membrane design and operating optimization, which is directly relevant to adaptive membrane control.

Digital twins can be used in water and wastewater treatment to track system performance, forecast fouling progression, and instantly improve operating conditions. To forecast membrane reactor efficiency from operational variables, Mahmoudi et al. [198] used artificial neural networks, where multilayer perceptron and radial basis function models were used to capture the nonlinear interactions between flow, pressure, rejection, and system performance. These features enable oxidative filtration to balance energy consumption, chemical demand, and removal efficiency while aligning with evolving regulatory goals. They also enable continuous tracking of conversion, oxidant utilization, and byproduct formation, as well as the prediction of when performance will fall below acceptable thresholds [199].

In practical oxidative filtration systems, these tools can support decision-making in several specific ways. For example, machine-learning models (e.g., artificial neural networks, random forest, support vector regression, gradient boosting, long short-term memory networks, or Gaussian process regression) can use real-time data such as flux, transmembrane pressure, conductivity, UV_254_, or TOC, oxidant residual, current density, and effluent pollutant concentration to predict flux decline, loss of oxidative activity, or excessive oxidant consumption before failure occurs. The digital twin can then recommend operating adjustments, such as reducing flux, changing oxidant dose, modifying current density, or initiating cleaning before irreversible fouling develops. At the design stage, the same framework can compare membrane structures or operating windows by linking pore size, active-layer thickness, charge density, and conductivity with predicted permeance, pollutant conversion, energy demand, and byproduct risk. Thus, AI and digital twins can improve both membrane design and plant operation by moving optimization from trial-and-error testing toward predictive and adaptive control.

**Figure 12:**
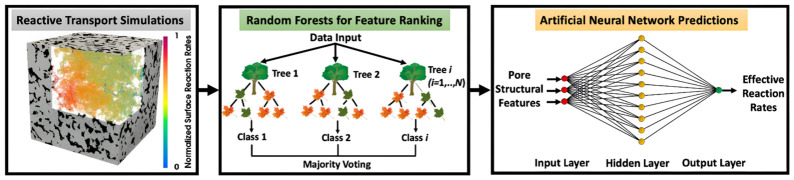
The schematic of the ML-based framework that combines 3D pore-scale reactive transport, Random Forests (RF), and Artificial Neural Network (ANN) models. Reproduced from Ref. [200], with permission from Nature.

When digital twins are connected to mechanical reactive transport models instead of being restricted to empirical prediction, they become particularly potent. Liu et al. [200] demonstrated that AI can meaningfully link microstructure and reactivity by first conducting pore-scale reactive transport simulations and then training machine-learning models to predict effective reaction rates from a limited set of pore structural parameters (Figure 12). This means that for oxidative filtration, digital twins could be used as both a monitoring tool and a design and control platform for intelligent membrane operation by combining sensor data with transport-reaction models to predict how membrane structure, fouling, and flow conditions affect reaction zones and long-term performance [201].

#### 9.2.2. Requirements for Oxidative Filtration Systems and Integration with Reactive Transport Models

To prepare oxidative filtration modules for digital twins, plants need to standardize their data architecture and sensing systems. Flow rate, pressure on both sides of the membrane, feed and permeate water quality parameters (e.g., conductivity, TOC, surrogate UV or oxidant absorbance), and oxidant dosage levels must all be continuously measured. For the model to consistently link operating conditions to performance trends, supporting infrastructure should log these variables at appropriate time scales and store them in standardized, time-stamped formats. Using hierarchical decoupling, JSON-LD-based descriptions, and graph-based topological assembly, Wu et al. [202] demonstrated how a modular digital-twin framework can be constructed from standardized component information. This is directly applicable to oxidative filtration modules, which will require interoperable data structures rather than isolated instrument streams.

Furthermore, the documentation of the membrane and process design must be readable by the digital twin. To enable the model to map sensor signals to actual zones and system components, information such as membrane area, module geometry, active-layer thickness, and approximate reaction-zone position should be stored. Because of this, structured data pipelines and metadata templates are becoming increasingly crucial: even high-quality sensor data remains challenging to convert into a usable digital representation. Gumus et al. [203] report a drinking-water digital twin as a representative example, as their XGBoost-SHAP framework could only provide trustworthy scenario testing and fouling prediction when important indicators such as DOC, UV_254_, SUVA, and fouling percentage were clearly linked to hydraulic and chemical loading conditions.

To replicate the movement and interactions of pollutants and reactive species inside the membrane system, digital twins also depend on precise reactive transport models. Effective reaction rates can be predicted from pore-scale structural features, which can then be incorporated into continuum-scale models of membrane reactors, as shown in recent work on machine-learning–augmented transport. This implies that, for oxidative filtration, intrapore diffusion, convective transport, and localized oxidation within the catalytic polymer layer may be described using the same framework used to estimate effective reaction rates in porous media. In order to predict effective reaction rates from a limited set of structural descriptors, Liu et al. [200] combined pore-scale reactive transport simulations with machine learning. Similarly, Kazakovtseva et al. [197] demonstrated that neural network surrogates trained on simulation data can quickly replicate membrane system behavior using dimensionless similarity criteria, as discussed above.

Digital twins can capture mechanistic connections, including residence time distribution, radical-mediated oxidation, and fouling-induced regime shifts, by connecting these physics-based models with operational data [204]. This is particularly useful when assessing new oxidative membranes, since the digital twin can replicate hypothetical scenarios, such as alterations in feed quality or oxidant dose, before the full-scale module is constructed. This use case is supported by the wastewater digital-twin study by Iqbal et al. [205]. They tested operating trade-offs under uncertainty and determined the hydraulic retention time and aeration conditions that controlled treatment efficiency across seasons by integrating calibrated process models into an interactive simulation environment.

Predictive maintenance, which can greatly increase the lifespan of oxidative filtration systems and minimize unscheduled downtime, is one of the most useful advantages of digital twins. Digital twins can predict when transmembrane pressure will exceed operating limits, when cleaning is required, or when reactive polymer degradation begins to reduce conversion efficiency by fusing real-time sensor data with data-driven or physics-guided models. To maintain mechanical integrity and catalytic activity, these forecasts can be used to plan maintenance activities, adjust cleaning intensity, and prevent over-washing [203]. Furthermore, digital twins can help separate the two causes of performance decline, as oxidative membranes are subject to both mechanical stress and chemical aging. For instance, machine-learning algorithms trained on past campaigns can suggest various remediation solutions based on the attribution of pressure rise to fouling accumulation or to structural changes such as compaction or pore collapse. As it is already the case in other industrial sectors where dependability and safety are crucial, this type of predictive maintenance is likely to become a standard requirement for advanced membrane systems in the long run. According to Khan et al. [195], digital twins are increasingly being combined with machine learning in additive manufacturing to enable predictive control and adaptive process optimization. The same reasoning holds for oxidative filtration modules, which must be continuously monitored, updated, and adjusted.

### 9.3. Life Cycle Assessment, TEA, and Circular Design

#### 9.3.1. Importance of LCA in Membrane Technologies

From raw material extraction and production through operation and end-of-life disposal, life cycle assessment (LCA) has become a key method for assessing the environmental impact of membrane technologies [206]. LCA assists in quantifying trade-offs among energy-intensive production, chemical (oxidant) consumption, and waste generation or recycling for oxidative filtration membranes, which must be operated under oxidizing conditions before being discarded. Reviews of membrane-based wastewater treatment and desalination show that LCA may pinpoint hotspots, including energy consumption, fouling-related chemical cleaning, and membrane manufacture, and it can direct better design decisions early in development [207]. In reality, LCA compels the field to explicitly account for upstream and downstream loads and go beyond efficiency-centric metrics (such as flow or elimination). This means that even an extremely efficient oxidative filtration system for intrinsic reactive polymer membranes may not be the best option for the environment if it has high manufacturing emissions, a limited lifespan, or a replacement rate that accelerates the buildup of plastic trash [208]. Thus, long-lasting, chemically resilient, and recyclable designs can be prioritized via early LCA screening during the concept stage.

Techno-economic analysis (TEA) supports LCA by translating reactive polymer membrane performance into actual deployment costs [209]. For oxidative filtration, TEA should include capital costs for membrane production, module construction, and system integration, as well as operating costs for energy demand, oxidant use, cleaning frequency, and membrane replacement. A membrane that performs well in laboratory tests may nonetheless be commercially unappealing if it requires high oxidant doses, frequent regeneration, or short service intervals. In this way, TEA addresses the financial side of the deployment question, while LCA addresses the environmental side, and together they offer a more complete sustainability assessment [210]. From an environmental sustainability standpoint, the most advantageous systems will be those that combine high pollutant removal with low energy input, minimal chemical consumption, long membrane lifetimes, and realistic end-of-life options such as reuse, recycling, or repurposing.

In practice, environmental and technical performance are strongly interconnected, and improvements in one metric may introduce limitations in another. For example, increasing catalytic-site density or oxidant dosage may improve pollutant conversion but can also reduce permeability, increase energy demand, accelerate fouling, or shorten membrane lifetime. Likewise, aggressive cleaning protocols may restore hydraulic performance while simultaneously promoting polymer aging, active-site loss, or chemical consumption. These trade-offs become even more significant during scale-up, where module geometry, flow distribution, pressure drop, residence time distribution, and cleaning accessibility strongly influence system behavior under continuous operation. Therefore, pilot- and large-scale evaluations should extend beyond short-term removal tests and include long-term stability, oxidant utilization, hydraulic productivity, byproduct formation, regeneration efficiency, maintenance frequency, and overall operational cost under realistic water matrices. Such an integrated assessment is necessary to determine whether high laboratory performance can be translated into sustainable and economically viable treatment systems.

#### 9.3.2. Lifetime, Durability, and Recycling Strategies for Oxidative Filtration Membranes

A longer membrane lifetime minimizes the need for replacement and distributes the environmental costs of manufacturing across a larger volume of treated water, making it a key metric in life cycle assessment. Durability must be assessed in actual oxidizing conditions for oxidative filtration, not merely under mechanical or pressure stress. According to studies on ordinary RO and NF membranes, their normal service lives are between five and seven years. LCAs based on these durations show that when membranes are retired early, the environmental impact is dominated by manufacturing and replacement. This implies that polymer-aging resistance under oxidative stress should be considered a crucial design criterion rather than merely a performance indicator for intrinsic reactive polymer membranes. In addition to reducing inventory replacement and related manufacturing emissions, a longer-life membrane also lowers the frequency of chemical cleaning and regeneration, both of which affect life cycle scores [211]. In this way, LCA offers a mathematical justification for funding redox-active and stable-radical chemistries that withstand deterioration under extended oxidant exposure [212].

Concurrently, end-of-life (EoL) membrane recycling and reuse are becoming essential elements of a circular membrane economy [213]. Compared with landfilling or incineration, direct recycling of reverse osmosis membranes into nanofiltration or ultrafiltration units can greatly reduce environmental impacts, particularly when the functional unit is treated water rather than the module itself, according to recent LCA-based and circular-economy reviews [214]. Similar methods have been proposed for downcycling high-pressure membranes into lower-pressure polishing stages and for upcycling low-pressure membranes (e.g., UF/MF) into higher-selectivity NF-like units. This shows that even modest reuse pathways can lessen the burden of solid waste and the potential for global warming. These techniques imply that spent catalytic membranes could be used for oxidative filtration rather than being discarded. For instance, the substrate could feed into NF/RO-to-NF recycling streams, or a reactive polymer layer that has lost oxidant sensitivity but maintains structural integrity could be repurposed in lower-value polishing or pre-treatment roles. These closed-loop solutions support the ‘paradigm shift’ from linear to circular membrane life cycles and offer a workable means of extending each membrane’s environmental benefits across multiple service phases while integrating lifetime and recycling considerations into the design process [215].

### 9.4. Future Integration: Toward Intelligent Treatment Systems

#### 9.4.1. Coupling AI + Digital Twins + Materials Design

The future of oxidative filtration will rest in the close integration of AI-driven materials design, digital twins, and real-time process control [216]. Recent reviews on hybrid models and digital twins for membrane systems show that merging physics-based simulations with machine learning offers rapid, accurate predictive tools that can capture fouling, energy use, and performance vs. cost trade-offs at both module and plant scales [217]. For oxidative filtration, this means that material descriptors (e.g., radical content, porosity, and transport-reactivity coupling) can be used to train models that estimate not only flow and removal but also aging and byproduct generation under realistic conditions. In this framework, experimental data continuously feed into digital twins, which in turn provide feedback to the design loop: for example, lab-scale kinetic and fouling data can be used to refine transport-reaction coefficients, while pilot-scale pressure-drop and conversion records can be used to adjust module-scale parameters. This makes a closed-loop design cycle in which new membranes are co-optimized with their operating envelope, rather than being tested in isolation and then adapted to the plant. For developing pollutants, such integrated procedures offer a means to build ‘smart barriers’ that are tailored to both chemical structure and hydrodynamic conditions.

#### 9.4.2. Autonomous Water-Treatment Systems

Autonomous or semi-autonomous treatment systems are emerging in wastewater and drinking-water infrastructure, where AI-enabled controllers use predictive models and real-time sensors to maintain water quality while reducing costs and energy use. Studies on model-predictive control and AI-driven WWTP operation reveal that automatic controllers can balance several objectives-such as effluent quality, sludge retention, and energy use-by modifying pumps, valves, and chemical dosing in response to influent variability [218]. In parallel, AI-driven management solutions are being utilized to optimize irrigation, detect defects, and offer operational interventions without constant human inspection [219]. For oxidative filtration, this envisions a future in which membranes are not static parts in a train but adaptive components in an intelligent treatment network. In such a picture, the membrane module would self-tune its flow, backwashing, and oxidant input based on feed-quality indicators, anticipated fouling, and regulatory restrictions. Physics-informed digital twins can ensure that the system remains within acceptable operating ranges while harnessing the full reactivity of the polymer, moving from fixed-setpoint operation toward adaptive, self-optimizing modes [220].

#### 9.4.3. Key Research Gaps

Despite tremendous improvement, numerous critical gaps remain before intelligent oxidative filtering systems can be applied at scale [199,218]. First, there is a dearth of high-quality, standardized datasets that link membrane chemistry, structure, and long-term performance across varied water matrices, which limits the generalizability of AI and digital-twin models. Second, many contemporary digital-twin frameworks for water treatment are constructed around traditional processes (e.g., ultrafiltration, activated sludge, or RO) and currently lack the EC-specific water-quality and reaction-pathway modules needed to accurately track oxidative filtration. Third, the field lacks widely established benchmarking methodologies for “AI- and DT-ready” membranes, including agreed-upon metrics for data reporting, sensor requirements, and uncertainty quantification. Closing these gaps will require coordinated efforts: establishing small-scale reference datasets, defining metadata templates for oxidative-filtration studies, and testing digital-twin prototypes in pilot plants operating under real-water conditions. Until these aspects are in place, most AI and digital-twin development will remain closer to proof-of-concept than to deployable, utility-grade control systems.

## 10. Grand Challenges and a Research Roadmap

Intrinsic reactive polymer membranes will not become a winning water-treatment paradigm by showing high degradation in idealized bench cells alone. They must demonstrate a system-level advantage over existing advanced treatment trains on five fronts simultaneously: stable transport, durable reactivity, credible mechanism, regulatory safety, and competitive cost. Full-scale ozonation already achieves broad micropollutant degradation at utility scale, and the revised EU urban wastewater framework now uses an average 80% removal benchmark for indicator micropollutants in quaternary treatment [221]. At the same time, membrane scientists are increasingly warning that materials discovery by itself has not translated into lower-cost or more deployable water-treatment technologies. The most plausible path forward is therefore not “better chemistry alone,” but a coordinated program that couples durable polymer design, standardized challenge matrices and ROS diagnostics, module-level hydrodynamics, long-duration pilot trials, and stage-gated TEA/LCA/digital-twin workflows. This outlook extends the pilot/TRL and AI-digitization logic developed in the preceding sections.

The targets summarized in Table 4 are proposed by the authors as synthesis milestones rather than fixed standards, but they are intentionally calibrated against current benchmarks from full-scale ozonation, pilot membrane reclamation, early electrified-membrane demonstrations, long-term electrochemical stability studies, and internationally recognized byproduct thresholds.

### 10.1. Key Barriers to Widespread Adoption

The decisive comparison is not against stirred-batch AOP kinetics but against incumbent multi-barrier trains already used for advanced water treatment. Bourgin et al. (2018) showed that full-scale ozonation at a specific dose of 0.55 g O_3_/g DOC achieved at least 80% average removal of selected indicator micropollutants [136], and Kienle et al. (2022) further showed that ozonation-based upgrading reduced multiple ecotoxicological endpoints by 66–93% without evidence of problematic reaction products [228]. In parallel, the revised EU framework for quaternary treatment uses a minimum average removal of 80% for at least six of twelve indicator compounds as a regulatory benchmark. Reactive polymer membranes must therefore show value beyond “single-compound >90% degradation in DI water”; they must deliver comparable broad-spectrum control under realistic matrices while consolidating separation and oxidation in a smaller footprint [136].

The first grand challenge is durability, because the field is still much closer to proof-of-concept than to utility-grade practice. Recent reviews of advanced functional and electrified membranes continue to identify cost, scalability, and long-term stability as the dominant bottlenecks, while McCutcheon and Mauter (2023) argue more broadly that membrane innovation fails when discovery is decoupled from manufacturability and operating reality [229]. A useful near-term gate is not a record removal value, but a real-matrix durability test: at least 100 h continuous operation with less than 10% loss in reactive flux or permeance and less than 20% decline in removal of representative indicators. Mid-term progress should require at least 1000 h module testing with repeated fouling and regeneration cycles; long-term field readiness should mean an equivalent service life of at least three years, even if the ultimate sector benchmark remains the 5–7-year replacement cycle, typical of RO/NF assets. Reactive membranes operate under harsher oxidative stress than passive membranes, so demanding the full RO lifetime at the outset may be unrealistic, but anything much shorter will likely fail TEA and LCA screens [230].

The second grand challenge is mechanistic certainty. ROS selectivity is central to the claimed advantages of these materials; however, several studies have highlighted that singlet oxygen is often over-assigned when relying solely on indirect scavenger-based evidence. Comprehensive analyses emphasize the need for orthogonal validation methods, as common ROS identification approaches can lead to misinterpretation of dominant reaction pathways [46]. The positive example remains the Janus electrocatalytic membrane of Zhao et al. (2020), where pore-confined ^1^O_2_ was supported by EPR, selective probes, and scavenger tests rather than by a single diagnostic [15]. For this paradigm to win, mechanistic claims should require at least two orthogonal diagnostics plus a kinetic consistency check; where feasible, steady-state ROS exposure or selectivity maps should be reported instead of merely naming a “dominant ROS.” Safety endpoints must be built into that same package: parent-compound removal is insufficient if bromate, halogenated byproducts, or ecotoxic transformation products rise. As a practical cross-jurisdictional reference point, bromate should remain below 10 µg L^−1^ wherever oxidant chemistry makes it relevant, even though site-specific discharge or reuse rules are otherwise unspecified.

The third grand challenge is realistic benchmarking. Hübner et al. (2024) explicitly called for comparable and scalable oxidation experiments [45], and Rath et al. (2024) showed that realistic ozonation behavior can be mimicked only when synthetic matrices reproduce the scavenging environment of real water [231]. More broadly, recent membrane studies on synthetic waste streams caution that oversimplified formulations distort fouling, kinetics, and even engineering-economic conclusions. For reactive polymer membranes, the community should adopt at least three benchmark matrices. A screening matrix should isolate the mechanism. A secondary effluent surrogate should include DOC, alkalinity, chloride, hardness, and a mixed micropollutant panel aligned where appropriate with current indicator compounds. A concentrate/brine matrix should stress salinity, scavenging, and byproduct pathways. Exact composition should remain chemistry- and application-specific and is therefore unspecified here, but it should be disclosed in full and reported using the minimum checklist in Table 2.

The fourth grand challenge is engineering scale-up. Performance gains observed at the lab scale, particularly those linked to pore-level confinement, often diminish once membranes are incorporated into full modules with realistic spacers, manifolds, and non-uniform flow distributions. Recent studies in electrochemical advanced oxidation highlight that computational fluid dynamics (CFD) has become essential for understanding how reactor geometry, flow fields, and confined transport influence ROS generation and mass transfer. Accordingly, scale-up must address both hydrodynamics and membrane chemistry, rather than treating materials optimization in isolation [232]. A practical TRL progression can be defined as follows: TRL 4 at 0.01–0.1 m^2^ with short-term testing in real matrices; TRL 5 at 0.1–1 m^2^ with CFD-guided module design and 100–500 h operation; TRL 6 at pilot scale (0.5–5 m^3^ h^−1^) for 1000–3000 h; and TRL 7 involving 10–100 m^3^ h^−1^ side-stream or full-train demonstration over 6–12 months. For pressure-driven systems such as NF-like membranes, a target reactive permeance of approximately 5–10 LMH bar^−1^ is reasonable. In contrast, porous catalytic or electrofiltration systems should aim for a stable flux of 20–50 LMH under continuous operation. For non-pressure-driven processes, performance should instead be reported in terms of residence time, charge per treated volume, and specific energy consumption, rather than pressure-normalized permeance.

The fifth grand challenge concerns economics, regulatory acceptance, and circularity. Pilot-scale water reuse studies already define the practical performance window. For example, long-term operation of a 1.5–2 m^3^ h^−1^ prototype over 18 months reported energy demands of approximately 0.35–1.3 kWh m^−3^ and operating costs of 0.18–0.31 € m^−3^, depending on configuration. In contrast, electrocatalytic membrane systems tested under simplified conditions have achieved much lower energy consumption (e.g., ~13.3 Wh m^−3^), highlighting the gap between controlled laboratory performance and real-world operation [15]. A realistic near- to mid-term target for reactive polymer membranes is therefore incremental energy consumption below ~0.3–0.5 kWh m^−3^ in polishing applications, with total operating costs competitive with existing quaternary treatment once cleaning, replacement, and byproduct management are included. For chemically driven systems, oxidant demand must be carefully normalized to treated volume and DOC. As a reference, processes should remain within the typical 0.4–0.6 g oxidant-equivalent per g DOC range observed in ozonation, or demonstrate a clear reduction relative to conventional AOPs at comparable removal efficiency. Importantly, TEA and LCA should be integrated from early development stages (TRL 3 onward). Without this parallel evaluation, there is a significant risk of advancing membrane technologies that perform well at the laboratory scale but are not economically viable, regulatory compliant, or sustainable at full scale [233].

### 10.2. Roadmap to Field Adoption

Industrial translation of reactive polymer membranes requires a roadmap that connects research gaps with measurable performance targets and technology-readiness challenges. The most critical gaps include long-term durability in real wastewater matrices, reliable ROS verification, standardized benchmark testing, scalable module hydrodynamics, regeneration without loss of oxidative function, and early TEA/LCA evaluation. These gaps should be assessed using practical metrics such as flux or permeance retention, pollutant conversion, oxidant utilization, byproduct formation, cleaning-cycle recovery, energy demand, and projected service lifetime. Table 4 summarizes these priorities and milestone-based targets to guide progress from laboratory validation toward pilot and full-scale deployment.

A systems-level roadmap is also needed because published studies often emphasize different performance endpoints, making direct comparison difficult. For example, some flow-through electrocatalytic membranes report high micropollutant removal and low energy demand under controlled laboratory conditions [15], while full-scale ozonation [136] and UV-AOP studies are usually evaluated by different criteria, such as DOC-normalized oxidant dose, EEO, byproduct formation, post-treatment needs, and long-term robustness [234]. Similarly, a membrane that achieves high removal in deionized water at low flux cannot be directly compared with a pilot-scale process operated for months in secondary effluent unless flux, residence time, matrix composition, oxidant utilization, energy demand, and stability are considered together. In this review, the proposed roadmap therefore refers to a staged evaluation pathway: first, mechanism and short-term performance are verified under controlled conditions; second, the same system is tested in benchmark and real-water matrices with standardized reporting; third, pilot modules are evaluated for hydraulic stability, cleaning recovery, byproduct control, TEA/LCA, and service lifetime. This integrated comparison helps identify whether a membrane is genuinely closer to deployment or whether its performance depends on favorable laboratory conditions, such as low flux, high oxidant dose, short operation time, or simplified water chemistry.

In the short term, the field should behave like an emerging platform technology rather than a collection of isolated materials papers. Academia should lead four tasks: build open descriptor sets for polymer chemistry, pore architecture, and ROS behavior; establish interlaboratory challenge matrices and ROS protocols; publish long-duration stability data; and create physics-guided substitute models that learn from pore-scale reactive-transport simulations. Industry should translate the most promising chemistries into scalable coating, casting, or interfacial-polymerization routes on module-compatible supports. Early engagement with regulators and standards bodies is critical to establish accepted frameworks for byproduct evaluation, leachate screening, and bioassay-based risk assessment prior to large-scale deployment. Consistent use of standardized reporting protocols (Table 2) is essential at this stage, as data-driven tools such as artificial intelligence and digital twins depend on high-quality, structured metadata for reliable model development [235]. Polymer informatics and AI-assisted design have reached a level of maturity that enables accelerated materials discovery and process optimization; however, their effectiveness is strongly dependent on the availability of curated datasets that incorporate mechanistic descriptors rather than isolated performance metrics.

In the mid-term, the focus transitions toward pilot-scale integration. Deployment of modular systems in the range of 0.5–5 m^3^ h^−1^, operated for 1000–3000 h under at least two representative water matrices, enables evaluation under realistic conditions [156]. Performance assessment extends beyond internal controls (e.g., no-voltage or no-oxidant operation) to direct comparison with established treatment trains such as ozonation followed by biofiltration or GAC, and NF/RO polishing. Key outcomes are expressed as operating envelopes rather than isolated optimum values, including relationships between flux or permeance and removal efficiency, energy demand and oxidant utilization, byproduct formation and selectivity, and cleaning intervals versus long-term performance loss [236]. At this stage, digital twin frameworks emerge as practical tools for monitoring and optimization. A minimum sensor set includes flow rate, transmembrane pressure, conductivity, UV_254_ or TOC proxies, electrical inputs (current/voltage), and oxidant residuals. These data streams support predictive models capable of identifying degradation in hydraulic performance or oxidative activity. In parallel, techno-economic and life cycle assessments are refined using pilot-scale data, incorporating uncertainty analysis and end-of-life considerations to provide a more accurate evaluation of system viability.

Over the long term, field adoption is most likely to begin in applications where reactive membranes provide clear system-level advantages. These include compact quaternary polishing under space constraints, treatment of NF/RO concentrates where contaminant enrichment enhances reaction efficiency, and decentralized or specialized systems where chemical supply is limited but electrified operation is practical. Progress toward deployment is supported by extended demonstration campaigns at scales of approximately 10–100 m^3^ h^−1^ or equivalent side-stream operation, with evaluation periods on the order of one year. Key performance indicators include sustained compliance with treatment targets, manageable maintenance intervals, and defined strategies for module replacement or recycling. Technology readiness ultimately depends on achieving sufficient durability and robustness under variable conditions. Limited operational lifetimes or strong dependence on narrowly defined feed compositions would restrict deployment to specialized or limited-use applications. In contrast, systems capable of maintaining at least 80% removal of indicator contaminants, low residual toxicity, competitive operating costs, and stable, data-driven process control present a credible pathway toward broader integration into water treatment infrastructure [237].

**Figure 13:**
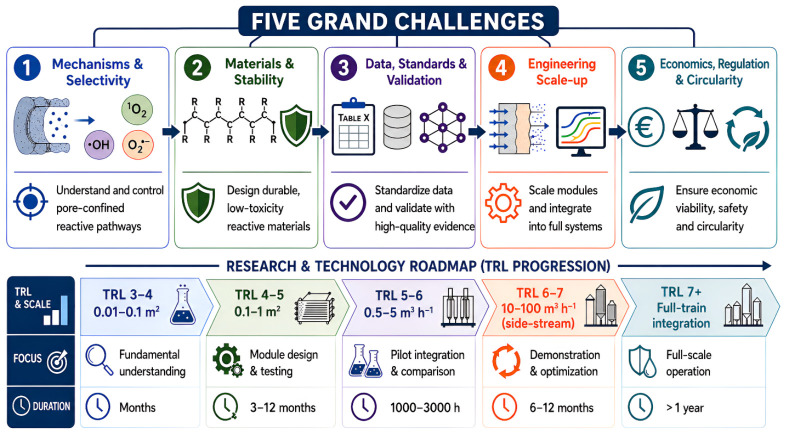
Schematic roadmap linking five grand challenges to technology readiness progression for reactive polymer membranes.

Figure 13 outlines the five grand challenges and the corresponding roadmap from fundamental research to full-scale deployment of reactive polymer membranes.

The overarching conclusion of this review is that reactive polymer membranes are best understood and developed as integrated treatment platforms, rather than as isolated material innovations. Progress in the field will depend on aligning advances in polymer chemistry with realistic operating conditions, robust mechanistic validation, and scalable engineering design. Priorities such as long-term stability, reliable identification of reactive pathways, hydrodynamic optimization at the module level, and consistent techno-environmental evaluation are essential for translating laboratory performance into practical systems. With sustained focus on these elements, current concepts can evolve into credible pilot-scale technologies and, ultimately, viable components of advanced water treatment infrastructure. In contrast, continued reliance on short-duration experiments in simplified matrices and incomplete mechanistic interpretation risks limiting progress to incremental material improvements without corresponding impact at the system level. Bridging this gap between fundamental science and application will determine whether reactive polymer membranes emerge as a transformative solution for water treatment or remain confined to proof-of-concept demonstrations.

## Figures and Tables

**Table 1:** Comparative summary of major classes of reactive polymeric membranes for oxidative filtration.

Membrane Class [REFS]	Main Reactive Mechanism	Key Advantages	Main Limitations	Scalability Outlook
Stable-radical polymer membranes [19,21]	Persistent radicals or oxygen-regenerated radical sites promote direct oxidation of pollutants during filtration.	Catalyst-free or metal-free operation; polymer itself provides reactivity; potential for simple membrane integration.	Radical stability may decrease over time; possible sensitivity to fouling, oxygen availability, and polymer aging.	Promising but early-stage; requires long-term stability testing, regeneration protocols, and larger-area fabrication.
Redox-active polymer membranes [23,25]	Redox groups such as quinone/hydroquinone, phenazine, or azo units mediate electron transfer and oxidant activation.	Tunable chemistry; can act as electron shuttle or co-catalyst; suitable for coupling with Fenton-like or peroxide-based systems.	Redox-site deactivation, leaching, and dependence on oxidant chemistry may limit durability.	Moderate potential; scalable if redox groups can be incorporated into robust commercial membrane formats.
Conductive polymer membranes [28]	Conductive polymers support electron transfer under applied potential, enabling electrochemical oxidation or reduction during flow-through operation.	Combines filtration and electrochemical treatment; controllable by voltage/current; potential for reagent-free operation.	Requires electrical integration; possible polymer over-oxidation, gas evolution, and energy-cost concerns.	Good potential for modular systems, but module design, electrode stability, and energy efficiency must be validated.
Porous conjugated polymer catalytic membranes [29,32]	Conjugated microporous or covalent polymer networks activate oxidants and control ROS selectivity through confined pores and functional sites.	High surface area; tunable pores and electronic structure; strong potential for selective ROS generation such as ^1^O_2_.	Fabrication can be complex; pore blockage, mass-transfer limits, and scale-up of thin catalytic layers remain challenges.	High scientific potential; practical deployment depends on scalable coating/casting methods and stable operation in real waters.

**Table 2:** Recommended minimum reporting checklist for oxidative filtration studies, including membrane characteristics, operational parameters, and performance metrics to ensure comparability and reproducibility across studies.

Parameter	Units/Details	Example	Notes	Reference
Membrane type	Material, MWCO/pore size, configuration	NF-90, ~200 Da	Include module type (flat sheet, hollow fiber)	[110]
Active/catalytic layer	Composition, thickness	TiO_2_-coated ceramic layer	Specify catalytic or reactive function	[111]
Clean-water flux	LMH ^1^ at defined pressure	120 LMH @ 5 bar	Baseline permeability	[116]
Reactive flux	LMH under operation	95 LMH @ 5 bar	Must match pressure/conditions	[109]
Permeance	LMH bar^−1^	24 LMH bar^−1^	Enables cross-study comparison	[117]
Influent pollutant(s)	µg/L or mg/L	Carbamazepine 5 µg/L	List all compounds tested	[118]
Water matrix	Composition	NOM ^2^ + salts	Must distinguish DI vs. real water	[119]
Oxidant type	Chemical/electrochemical	H_2_O_2_, PMS, current	Clearly define source	[120]
Oxidant dose	mg/L or mM	20 mg/L H_2_O_2_	Normalize to treated volume	[87]
Current density	mA/cm^2^	20 mA/cm^2^	For electrochemical systems	[112]
Charge per volume	C/L or kAh/m^3^	30 C/L	Standard electrochemical metric	[88]
Dominant ROS	Identified species	HO^•^ via TPA	Must include validation method	[121]
Removal efficiency	% and concentration	95%, 10→0.5 µg/L	Report both forms	[122]
Byproducts	Identified compounds	Oxalic acid, bromate	List key products	[123]
Byproduct analysis method	LC-MS/GC-MS	LC-MS/MS	Include detection limits	[114]
Processed volume	L or m^3^	5 L	Important for scaling	[124]
Flow rate	mL/min or LMH	10 mL/min	Include flow regime	[116]
Cleaning method	Chemical/physical	NaOH + backwash	Describe protocol	[125]
Feed composition	Full matrix	NOM + Ca^2+^ + Cl^−^	Must be fully reported	[126]

^1^ Liters per square meter per hour; ^2^ Natural organic matter.

**Table 3:** Representative performance ranges and practical considerations for membrane-based, oxidative, electrochemical, and hybrid treatment processes, including typical micropollutant removal, hydraulic productivity, energy demand, main advantages, and key limitations relevant to utility-scale deployment.

Process/System	Main Function	Typical Micropollutant Removal	Typical Flux/Permeance	Typical Energy Demand	Main Advantages	Main Limitations	References
UF/MF/MBR	Solids, biomass, and macromolecule retention	Low–moderate; often incomplete for dissolved micropollutants	~10–100 LMH	~0.3–1.0 kWh m^−3^	Mature, compact, good biological integration	Limited removal of small dissolved contaminants; fouling	[152,153]
NF	Size/charge-based rejection of small organics and ions	~50–>95%, compound-dependent	~5–30 LMH bar^−1^	~0.5–2.5 kWh m^−3^	High rejection, useful for polishing	Concentrate generation; fouling; pressure demand	[154]
RO	High-retention desalination and micropollutant rejection	Often >90–99%	~1–5 LMH bar^−1^	~1–6 kWh m^−3^	Strong barrier for salts, PFAS, and many micropollutants	Brine management; high pressure; scaling/fouling	[155]
Ozonation + biofiltration	Oxidation followed by biodegradable byproduct removal	~70–95%, depending on ozone dose and compound	Not membrane-limited	~0.05–0.5 kWh m^−3^ equivalent; oxidant dose often normalized to DOC	Mature quaternary treatment; broad micropollutant abatement	Bromate/byproduct risk; matrix-dependent oxidant demand	[156,157]
UV/H_2_O_2_ AOP	Radical-based oxidation	~70–>99%, compound- and dose-dependent	Not membrane-limited	EEO often ~0.1–10 kWh m^−3^ order^−1^, but highly variable	Strong oxidation; established design basis	High energy/chemical demand; scavenging by NOM and carbonate	[66,151]
Electrochemical/conductive membrane filtration	Flow-through electrochemical oxidation/reduction	~60–>95% in reported lab systems	Often ~10–100 LMH	From Wh m^−3^ to kWh m^−3^, depending on cell design and water conductivity	Reagent-free or low-chemical operation; controllable by current/voltage	Electrode aging, gas evolution, conductivity dependence, scale-up	[26,158]
Reactive polymer oxidative membranes	Combined filtration, enrichment, and in situ degradation	~50–>95% in early studies	Highly variable; often ~10–100 LMH or reported as permeance	Not yet standardized; report EEO, charge dose, or oxidant dose	Compact treatment; simultaneous separation and degradation	Early TRL; stability, byproducts, ROS verification, and real-matrix testing needed	[20,159]
Adsorption–oxidation hybrid membranes	Pollutant enrichment followed by local oxidation/regeneration	Often high for adsorbing compounds; matrix-dependent	Depends on support and adsorbent loading	Depends on oxidant/electrical input	Can improve local reaction rate and reduce secondary waste	Sorbent saturation, regeneration efficiency, byproduct control	[160,161]

**Table 4:** Grand challenges and milestone-based roadmap for the development and deployment of intrinsic reactive polymer membranes, outlining key priorities, performance targets across time scales, and recommended methods and stakeholders to guide translation from laboratory research to full-scale water treatment applications.

Priority	Grand Challenge	Short-Term Milestone	Midterm Milestone	Long-Term Metrics	Suggested Methods and Lead Stakeholders	REFS
Very high	Durability of reactive polymers and active layers	≥100 h continuous operation; <10% flux/permeance loss; <20% drop in removal	≥1000 h module test with cleaning/regeneration; low delamination; acceptable leaching limits	≥3 years service life; annual activity loss <20%; end-of-life route defined	Accelerated oxidative aging, cyclic fouling/cleaning, membrane autopsy, and spectroscopic/morphological analysis (XPS, FTIR, SEM) with leachate testing. Lead: academia + industry	[99,222]
Very high	Mechanistic certainty and ROS selectivity	Orthogonal ROS identification in a benchmark matrix (probe, scavenger, EPR)	Quantified ROS exposure/selectivity map across pH, NOM, alkalinity, and ionic strength	Mechanism-based operating envelope embedded in control strategy; byproducts predictably bounded	Kinetic modeling, isotope/labeling where feasible, probe validation, transformation-product, and bioassay analysis. Lead: academia + analytical labs	[46,223]
Very high	Realistic benchmark testing and reporting	Community challenge matrices and metadata template aligned with Table 2; interlaboratory round robin	Adoption by major research groups and pilot projects; open benchmark dataset	Standards-like reporting norm for the field	Standardized water matrices, round-robin testing, and FAIR data. Lead: academia + standards bodies + funders	[224]
High	Module hydrodynamics and scale-up	Reactive-area scale-up to 0.1–1 m^2^ with low pressure drop; <15% flow maldistribution	Pilot skid at 0.5–5 m^3^ h^−1^ for 1000–3000 h	Demonstration unit at 10–100 m^3^ h^−1^ or utility side-stream for 6–12 months	CFD, RTD analysis, pressure mapping, and flow-field optimization. Lead: industry + academia + utilities	[225]
High	Process integration, fouling control, regeneration	Standard foulant recipe and recovery protocol with flux and activity recovery	Cleaning/regeneration strategy validated over ≥20 cycles	Scheduled maintenance with predictable recovery and low chemical use	NOM/biopolymer/mineral fouling tests, oxidative vs. non-oxidative cleaning, flux, and activity recovery. Lead: utilities + industry	[92,226]
High	TEA, LCA, and regulatory acceptance	Preliminary TEA/LCA and hazard screen at TRL 3–4	Pilot-validated TEA/LCA with uncertainty analysis and permitting dossier	OPEX-competitive with quaternary treatment; byproduct compliance and safety acceptance	Parallel TEA/LCA with uncertainty analysis, byproduct monitoring, and recycling planning. Lead: industry + utilities + regulators	[227]

## Data Availability

No new data were created or analyzed in this study. Data sharing is not applicable to this article.
